# Explainable deep learning for healthcare workforce attrition: a methodological study on the Watson healthcare synthetic benchmark

**DOI:** 10.3389/fpubh.2026.1861450

**Published:** 2026-06-12

**Authors:** Dan Yin, Xinyu Lu, Tingyi Mei

**Affiliations:** Zhongshan Second People's Hospital, Zhongshan, Guangdong, China

**Keywords:** deep neural network, explainable artificial intelligence, healthcare employee retention, job satisfaction, workforce attrition prediction

## Abstract

**Background:**

Retaining qualified nurses and allied-health staff has become a central occupational-health concern for modern hospital systems, yet existing attrition-prediction models face a persistent trade-off between predictive accuracy and auditability. The quantitative contribution of modifiable occupational factors—working hours, work environment, compensation, and commuting burden—to individual departure decisions remains insufficiently characterized.

**Methods:**

We developed an end-to-end interpretable deep-learning framework and evaluated it on the publicly available *Watson Healthcare Employee Attrition* benchmark, a synthetic dataset derived from the IBM Watson HR Analytics dataset in which job roles and departments were relabeled to reflect the healthcare domain and a subset of outcome labels was modified to facilitate machine-learning evaluation; we therefore use the dataset strictly as a methodological benchmark rather than as a record of any real hospital workforce. The benchmark contains 1, 676 synthetic employee records described by 40 engineered features spanning demographics, work context, satisfaction, and compensation. A compact 40–128–64–32–2 multilayer perceptron was trained on a Synthetic Minority Over-sampling Technique (SMOTE)-balanced training set with AdamW optimization, cosine-annealing learning-rate scheduling, and early stopping. It was then interpreted using a DeepExplainer-based SHapley Additive exPlanations (SHAP) module operating on the log-odds margin. Performance was evaluated using 5-fold stratified cross-validation and benchmarked against logistic regression, random forest, and gradient-boosting classifiers.

**Results:**

The proposed network achieved an area under the receiver-operating-characteristic curve of 0.934 ± 0.026 and an area under the precision–recall curve of 0.761 ± 0.091, matching or exceeding all three baselines while preserving minority-class sensitivity. Dual-level SHAP analysis consistently identified overtime status, environment satisfaction, marital status, job involvement, tenure in current role, age, and commuting distance as the dominant drivers of the predicted attrition label on this benchmark. It revealed interpretable non-linear thresholds together with auditable, case-by-case rationales.

**Conclusion:**

On this synthetic benchmark, a compact interpretable deep network matches strong tabular baselines while providing axiomatic, dual-level SHAP explanations that translate into three candidate retention levers-overtime reduction, working-environment improvement, and commuting support. Because the records are synthetic, these levers should be regarded as data-driven hypotheses rather than validated interventions, and prospective evaluation on real, de-identified hospital workforce data remains a necessary next step.

## 1 Introduction

The sustained retention of qualified healthcare personnel has become one of the most pressing operational challenges facing modern hospital systems (1). Driven by the long-term consequences of population aging, accumulated workforce stress from global public health emergencies, and the chronic mismatch between demand for clinical services and the supply of trained nurses and allied-health professionals, voluntary attrition rates in hospitals have reached levels that directly threaten patient safety, continuity of care, and institutional financial stability (2). Unlike turnover in many other industries, the departure of a single experienced nurse entails costs that extend far beyond recruitment and onboarding: it disrupts established care teams, erodes tacit clinical knowledge accumulated over years of bedside practice, increases the cognitive and physical burden on the remaining staff, and, in the most consequential cases, measurably degrades patient outcomes (3). Against this backdrop, hospital human resources departments and nursing management offices are increasingly expected to move from reactive, exit-interview-driven retention strategies toward proactive, data-driven identification of at-risk employees and the evidence-based design of targeted interventions (4). The growing availability of structured personnel records—encompassing demographic attributes, work-context variables such as overtime intensity and commuting distance, satisfaction indicators along several psychosocial dimensions, and compensation attributes including monthly income, salary hike, and job level, together with recent advances in machine learning—creates a concrete opportunity to address this need (5). In particular, parallel advances in deep neural networks for heterogeneous tabular data and in axiomatic post-hoc explanation methods such as Shapley Additive exPlanations (SHAP) make it feasible, for the first time, to combine the predictive capacity needed to detect subtle, non-linear attrition patterns with the auditability required for clinical decision-making (5). Motivated by this conjunction of managerial urgency, methodological maturity, and the availability of standardized public benchmarks, the present study aims to develop and characterize, on the publicly available *Watson Healthcare Employee Attrition* benchmark—a synthetic dataset derived from the IBM Watson HR Analytics dataset with healthcare-relabeled job roles, used here as a methodological testbed rather than as a record of any real hospital workforce—an interpretable deep-learning framework that not only attains competitive predictive accuracy but also reveals, at both the dataset-wide and individual-record levels, the specific role played by job-satisfaction and compensation features in the dataset's attrition label (6). Recent independent studies, including the study of Marjerison et al. (26), have used the same benchmark in a similar methodological role; we engage with that line of work in Section 2.2.

Despite the rapid growth of machine-learning-based attrition research, three important gaps remain unresolved in the literature and motivate the specific contributions of this work (7). First, prior studies on employee attrition prediction have predominantly relied either on classical linear models, whose restricted functional form cannot accommodate the threshold-like and interaction-driven patterns that pervade real personnel data, or on tree-based ensembles, whose built-in feature-importance scores are not grounded in any axiomatic attribution framework and therefore cannot provide the local, per-employee rationales required for human-resource auditing; a compact, well-regularized deep network explicitly coupled with a Shapley-consistent explanation module remains comparatively underexplored in this application domain (8). Second, although job satisfaction and compensation are widely acknowledged in organizational-behavior and labor-economics research as central determinants of turnover, their *quantitative* magnitudes, *non-linear* operating ranges, and *case-by-case* manifestations in healthcare-specific cohorts have so far been assessed primarily through aggregate regression coefficients, leaving open the question of how these factors interact with workload, demographic, and commuting variables to drive individual departure decisions (9). Third, existing SHAP-based attrition studies tend to stop at global bar-chart rankings of feature importance and rarely exploit the full dual-level diagnostic potential of Shapley values, so that the translation from statistical attribution to concrete, ward-level retention interventions has remained largely informal. To close these gaps, the present study makes the following three contributions:

(1) We designed an end-to-end framework that couples a compact 40-128-64-32-2 multilayer perceptron—trained on a SMOTE-balanced healthcare-attrition benchmark with AdamW, cosine-annealing learning-rate scheduling and early stopping—with a DeepExplainer-based SHAP module operating on the log-odds margin. We demonstrate through 5-fold stratified cross-validation that this interpretable deep model attains predictive performance within one cross-fold standard deviation of strong tabular baselines including logistic regression, random forest and gradient boosting (under a leak-free cross-validation protocol; see Sections 4.4 and 4.5).
(2) Through a dual-level SHAP analysis combining dataset-wide beeswarm summaries, feature-level dependence plots and individual-level waterfall decompositions, we quantitatively characterized the role of job-satisfaction and compensation features—together with overtime status, marital status, tenure in current role, age and commuting distance—in driving the predicted attrition label on the benchmark, including their non-linear operating thresholds and their direction of effect on the predicted log-odds, thereby producing a transparent, axiomatically grounded characterization of the model's decision logic.
(3) We translated the resulting global and local attribution patterns into three candidate, evidence-based retention levers—overtime reduction, working-environment improvement, and commuting support—which are consistent with prior findings in the organizational-behavior literature on real-world workforces and which we therefore propose as *data-driven hypotheses for managerial action*. We emphasize that any clinical or human-resource deployment of these levers must be preceded by validation on real, prospectively collected hospital data; the contribution of the present study is the analytical template, not a validated decision tool.

## 2 Related work

### 2.1 Machine learning for employee attrition prediction

Quantitative studies of voluntary turnover have evolved along a clear methodological trajectory (10). Early work relied on classical statistical models—most notably binary logistic regression and Cox proportional hazards survival analysis—which provided interpretable closed-form coefficients but typically delivered modest predictive accuracy on high-dimensional, heterogeneous personnel data (11). The shift toward data-driven modeling over the past decade has introduced progressively richer function classes, including decision trees, random forests, support-vector machines, and gradient-boosting ensembles, whose non-linear decision boundaries proved particularly well suited to the mixed binary, ordinal, and continuous attributes that populate human-resource datasets. More recently, fully connected deep networks, autoencoder-based embeddings, and tabular transformer variants have been explored as additional capacity-expanding alternatives. Across this body of work, two recurring difficulties persist (12). The first is a pronounced class imbalance between stayers and leavers, which has been mitigated through minority oversampling, cost-sensitive training, or rebalanced loss formulations (13). The second is a systematic trade-off between predictive performance and model transparency, which has so far limited the operational adoption of high-capacity non-linear models in human-resource decision-making pipelines where auditability is a prerequisite (14).

### 2.2 Healthcare workforce retention

A parallel line of research focuses on retention dynamics in the healthcare sector, where staff turnover imposes elevated costs for recruitment, onboarding, patient safety, and continuity of care (15). Prior descriptive and regression-based analyses of nursing and allied health cohorts have consistently identified a set of recurrent antecedents of attrition, including job satisfaction, perceived organizational climate, work-life balance, compensation fairness, overtime intensity, commuting burden, career-progression opportunities, and demographic factors such as age and marital status (16). The existing literature, however, remains methodologically fragmented: many studies are survey-based and limited to small, single-institution cohorts, while larger-scale analyses typically employ linear or logistic models whose restricted functional form cannot capture the threshold-like and interaction-driven patterns that emerge in real-world workforce data (17). As a result, although the qualitative drivers of nurse attrition are well documented, their quantitative magnitudes, non-linear operating ranges, and case-by-case manifestations remain insufficiently characterized, leaving a gap that modern, interpretable machine learning is particularly well placed to fill (18).

### 2.3 Explainable artificial intelligence and SHAP-based attributions

Explainable artificial intelligence has emerged as a necessary bridge between black-box predictive performance and the accountability requirements of regulated decision environments such as medicine, finance, and human resource management (19). *Post-hoc* attribution methods—including perturbation-based local surrogates, integrated gradients, layer-wise relevance propagation, and class-activation-style approaches—provide instance-level rationales that domain experts can inspect without retraining the underlying model (20). Within this family, SHAP has become a *de facto* standard for tabular predictors because its grounding in cooperative game theory endows it with desirable uniqueness properties, namely local accuracy, consistency, and missingness (21). A growing body of work has applied SHAP to attrition prediction, most often on top of tree-based ensembles, and has reported global feature rankings that align with managerial intuition (22). Deep-learning-specific variants such as DeepExplainer and KernelExplainer also enable attribution of predictions from neural networks, thereby combining the representational capacity of deep models with the axiomatic transparency of Shapley attributions (23). Nevertheless, prior SHAP-based attrition studies frequently stop at global bar-chart rankings and rarely exploit the full dual-level diagnostic potential of Shapley values—namely the combination of cohort-level beeswarm summaries, feature-level non-linear dependence patterns, and individual-level waterfall decompositions that together enable audit-ready, case-by-case clinical reasoning (24).

### 2.4 Job satisfaction, compensation and retention determinants

A long tradition in organizational behavior and labor economics has examined the determinants of voluntary turnover, with job satisfaction and compensation occupying a central role (25). Classical theoretical frameworks posit that intrinsic satisfaction with the work environment, perceived fairness of monetary rewards, job involvement, and opportunities for career progression are primary drivers of retention, whereas extrinsic factors such as commuting distance, travel frequency, and family status exert a modulating influence. Empirical investigations, both general and healthcare-specific, have repeatedly corroborated these mechanisms at the aggregate level, yet quantitative assessments of their *relative* magnitudes, *non-linear* operating ranges, and *interactions* with demographic and work-context variables remain comparatively scarce (26). This gap is particularly acute in the nursing and allied health context, where shift-based scheduling, the prevalence of overtime, and compensation structures differ markedly from those in other industries and therefore cannot be reliably extrapolated from generic turnover studies (27).

### 2.5 Positioning of the present work

Taken together, the three streams above motivate but leave open the central problem addressed in this study: how to combine the predictive capacity of modern deep networks, the axiomatic transparency of Shapley-value explanations, and the domain specificity of healthcare workforce analysis within a single, reproducible, and clinically actionable framework (28). Our contribution is best characterized as a methodological synthesis along three axes (29). First, a compact, carefully regularized multilayer perceptron is trained on a SMOTE-balanced healthcare cohort under a 5-fold stratified cross-validation protocol, which yields predictive performance within one cross-fold standard deviation of strong tabular baselines such as logistic regression, random forests and gradient boosting (30). Second, the trained network is interpreted simultaneously at the global and individual levels using SHAP attributions computed on the log-odds margin, with explicit emphasis on non-linear dependence patterns and auditable waterfall decompositions of high- and low-risk employees. Third, the resulting attribution patterns are translated into three concrete, ward-level retention interventions—overtime reduction, working-environment improvement, and commuting support—thereby closing the loop between raw personnel records and evidence-based managerial actions. This loop is only partially closed in prior healthcare attrition studies.

## 3 Methodology

### 3.1 Model architecture design

**Figure 1:**
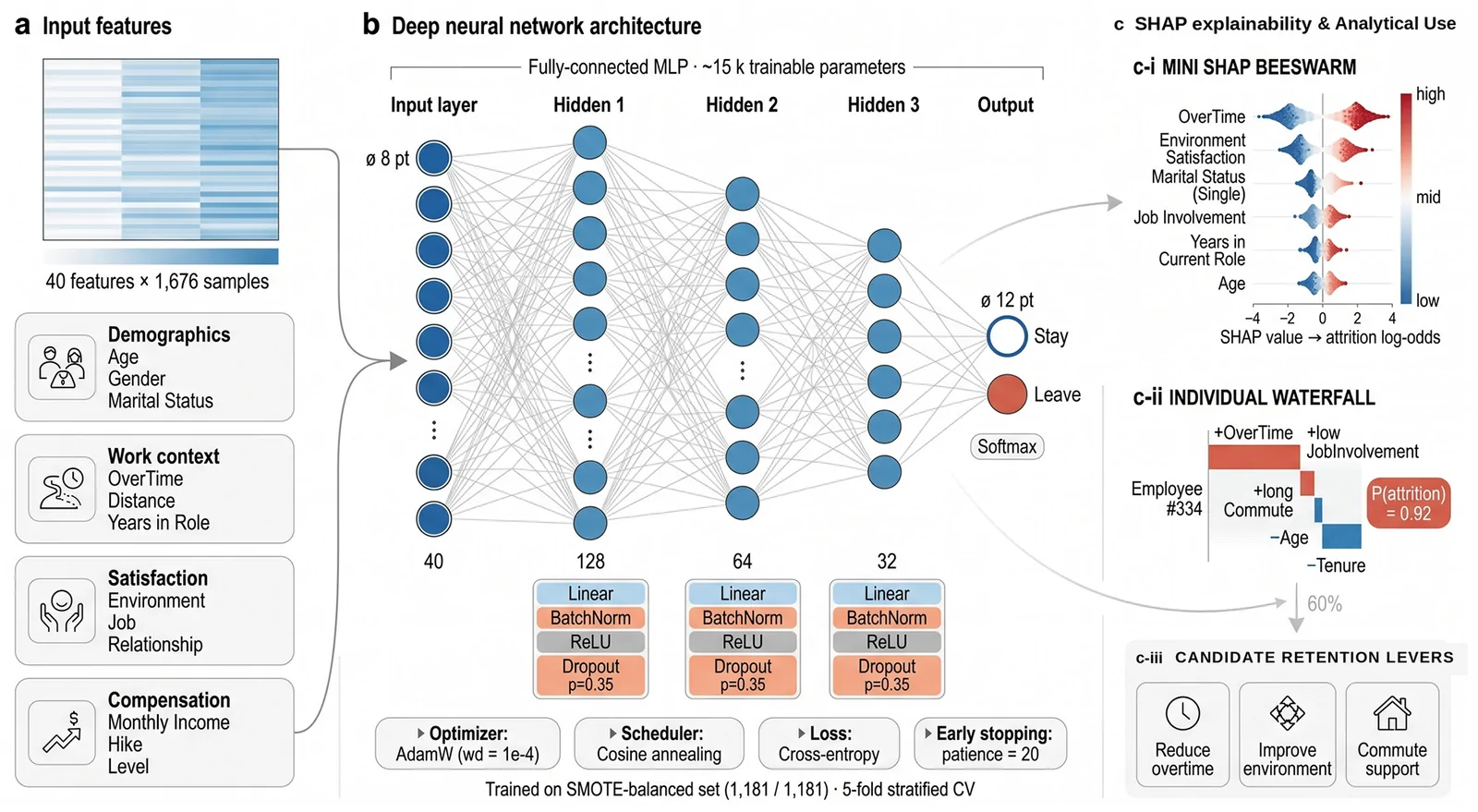
Overall framework of the SHAP-interpretable deep neural network for healthcare employee-attrition benchmark analysis. **(a)** Input features: a benchmark dataset of 1,676 synthetic employee records is represented by 40 engineered variables spanning demographics, work context, satisfaction, and compensation domains. **(b)** Deep neural network architecture: a fully connected multilayer perceptron with a 40–128–64–32–2 funnel topology (approximately 15 k trainable parameters); each hidden block combines Linear, BatchNorm, ReLU, and Dropout (*p* = 0.35) operations, followed by a softmax output that discriminates “Stay” from “Leave.” The network is trained on a SMOTE-balanced set (1,181/1,181) with AdamW (weight decay = 1 × 10^−4^), cosine-annealing learning-rate scheduling, cross-entropy loss, and early stopping (patience = 20), and is evaluated under 5-fold stratified cross-validation. **(c)** SHAP explainability and analytical use: **(c-i)** a mini beeswarm plot summarizes global feature contributions to the attrition log-odds; **(c-ii)** an individual-level waterfall plot decomposes a single record's predicted probability into positive and negative drivers; **(c-iii)** the resulting insights are translated into candidate retention levers—overtime reduction, environment improvement and commuting support—formulated as data-driven hypotheses for managerial action rather than as clinically validated interventions.

To jointly achieve competitive predictive accuracy and methodologically sound interpretability on the publicly available *Watson Healthcare Employee Attrition* synthetic benchmark, we designed an end-to-end framework that couples a compact deep neural network with a post-hoc SHAP explanation module, as shown in Figure 1. The pipeline comprises three tightly connected stages—feature construction, representation learning, and explanation-driven analysis—each of which directly corresponds to one panel of the figure.

The input stage (Figure 1a) ingests the 1, 676 synthetic employee records of the benchmark, described by 40 engineered features. After removing constant and identifier columns, applying binary encoding to two-level categorical variables, and performing one-hot expansion of multi-level categorical variables, the resulting feature matrix spans four semantically coherent domains: record-level *demographics* (e.g., age, gender, marital status), *work context* (e.g., overtime status, commuting distance, years in current role), *satisfaction* indicators (environment, job, and relationship satisfaction), and *compensation* attributes (monthly income, salary hike, and job level). All continuous features are standardized using a training-set *z*-score transform to stabilize optimization, and class imbalance is addressed with Synthetic Minority Over-sampling (SMOTE) applied strictly to the training fold to yield a perfectly balanced positive–negative set of 1,181 / 1,181 samples, thereby preventing any information leakage from the held-out test partition.

The representation-learning stage (Figure 1b) is a fully connected multilayer perceptron with a funnel topology of 40–128–64–32–2 units, amounting to roughly fifteen thousand trainable parameters. Each hidden block consists of a linear transformation, one-dimensional batch normalization, a rectified linear activation, and a dropout layer with a retention probability of 0.35; this ordering was chosen so that batch normalization stabilizes pre-activation statistics across mini-batches, the non-linearity provides the representational capacity required for the heterogeneous tabular inputs, and dropout provides stochastic regularization that mitigates the overfitting tendency associated with the modest sample size of the benchmark (*N* = 1, 676). The two-unit output layer is followed by a softmax transformation that maps the learned representation to a posterior probability over the binary outcome “Stay” vs. “Leave.” The network is optimized with AdamW (weight decay = 1 × 10^−4^) coupled to a cosine-annealing learning-rate schedule, minimizing a cross-entropy objective with early stopping triggered after twenty epochs without improvement on a held-out validation split. To provide unbiased performance estimates, the entire training protocol is further embedded in a 5-fold stratified cross-validation scheme, within which SMOTE and standardization are re-fitted on each training fold.

The explanation stage (Figure 1c) turns the trained network into a transparent analytical tool whose decisions can be audited at both global and individual-record levels. A DeepExplainer is used to attribute each prediction to its input features in the additive log-odds space of attrition. At the dataset-wide level, a summary beeswarm plot (Figure 1c–i) shows which behavioral and contextual drivers—such as overtime, environment satisfaction, marital status, job involvement, tenure in current role, and age—contribute most strongly to the predicted attrition score across the benchmark. At the individual level, a waterfall plot (Figure 1c-ii) decomposes a single record's predicted probability into additive positive and negative contributions, allowing case-level risk to be traced back to specific working conditions. These two complementary views are finally translated into candidate retention levers (Figure 1c-iii)—overtime reduction, working-environment improvements, and commuting support—that we propose as data-driven hypotheses for managerial action; we underscore that any deployment of these levers in a real hospital setting should be preceded by validation on real, prospectively collected workforce data.

### 3.2 Data preprocessing and class imbalance handling

#### 3.2.1 Dataset provenance and disclosure

The benchmark used throughout this study is the publicly available *Watson Healthcare Employee Attrition* dataset distributed via the Kaggle data repository (contributor: J. P. Miller). For full transparency, and following the explicit description provided by the dataset's contributor, we record the following four characteristics: (i) the data are *synthetic*, not collected from any real hospital workforce; (ii) the dataset is *derived* from the *IBM Watson HR Analytics Employee Attrition & Performance* dataset, itself a synthetic dataset created by IBM data scientists; (iii) the original IBM dataset is generic cross-sectoral HR data, and the healthcare framing of the present benchmark is obtained by *relabeling* job roles and departments to healthcare-domain values; (iv) a subset of the original outcome labels was *modified* relative to the IBM source to facilitate machine-learning evaluation. We therefore used the dataset strictly as a public, reproducible *methodological benchmark* for tabular attrition prediction and explainability research, consistent with prior methodological research on the same dataset (e.g., (26)); none of the numerical results reported in this study should be interpreted as describing real nurses, real allied-health professionals, or any real hospital workforce. The rest of the study, we accordingly refer to the 1, 676 rows of the benchmark as *synthetic employee records* (or simply *records*) rather than as a clinical cohort, and we frame all attribution findings as properties of the model on this benchmark, not as findings about real personnel.

The benchmark dataset described above contains 1, 676 synthetic employee records together with a binary attrition label (1= Leave, 0= Stay). Four structurally non-informative columns (EmployeeID, EmployeeCount, Over18, and StandardHours) are removed because they are either unique identifiers or constants, after which two strictly binary attributes (Gender and OverTime) are integer-encoded and five multi-level categorical attributes (BusinessTravel, Department, EducationField, JobRole, and MaritalStatus) are one-hot encoded with the first level dropped to avoid perfect collinearity. Denoting by *c* ∈ {1, …, *K*_*v*_} the categories of a variable *v* with baseline level *c* = 1, the encoding map is as follows:


$$\begin{array}{l} \text{onehot}\left(v=c\right)={\left(⊮\left[v=2\right],⊮\left[v=3\right],\ldots ,⊮\left[v=K_{v}\right]\right)}^{⊤}\\ \;\in {\left\{0,1\right\}}^{K_{v}-1}, \end{array}$$ (1)

This yields a design matrix **X** ∈ ℝ^*N*×*d*^ with *d* = 40 features across four semantically coherent domains: demographics, work context, satisfaction, and compensation. To stabilize gradient-based optimization of the deep network, every continuous feature is standardized to zero mean and unit variance using only the training-partition statistics.


$$\begin{array}{lll} \mu_{j}^{\text{tr}} & = & \frac{1}{N_{\text{tr}}}\underset{i\in I_{\text{tr}}}{\sum }x_{ij},\;\sigma_{j}^{\text{tr}}=\sqrt{\frac{1}{N_{\text{tr}}-1}\underset{i\in I_{\text{tr}}}{\sum }{\left(x_{ij}-\mu_{j}^{\text{tr}}\right)}^{2}},\\ {\tilde{x}}_{ij} & = & \frac{x_{ij}-\mu_{j}^{\text{tr}}}{\sigma_{j}^{\text{tr}}}, \end{array}$$ (2)

where ℐ_tr_ is the index set of the training fold, thereby preventing any information leakage from the held-out test partition. The cohort is then split into a stratified 80%/20% train/test partition that explicitly preserves the minority prevalence.


$$\begin{array}{l} \frac{\#\left\{i\in I_{\text{tr}}:y_{i}=1\right\}}{\left|I_{\text{tr}}\right|}=\frac{\#\left\{i\in I_{\text{te}}:y_{i}=1\right\}}{\left|I_{\text{te}}\right|}=\pi_{+}, \end{array}$$ (3)

where π_+_≈0.12 is the overall attrition rate, so that both subsets are statistically representative of the full benchmark.

Employee attrition is an intrinsically imbalanced outcome. With a class-imbalance ratio


$$\begin{array}{l} \text{IR}=\frac{N_{-}}{N_{+}}\approx \frac{1477}{199}\approx 7.4, \end{array}$$ (4)

which is a plain cross-entropy classifier, is systematically biased toward predicting the majority *Stay* class and therefore under-detects the clinically important minority. To counter this bias, we adopt the Synthetic Minority Over-sampling Technique (SMOTE), which generates synthetic minority samples along the line segments joining each minority instance to its *k* = 5 nearest minority neighbors under the Euclidean metric.


$$\begin{array}{l} N_{k}\left({\text{x}}_{i}\right)=\underset{\begin{array}{c} S\subseteq I_{+}\backslash \left\{i\right\}∥|S|=k \end{array}}{\mathrm{argmin}}\underset{j\in S}{\sum }||{\text{x}}_{i}-{\text{x}}_{j}|{|}_{2}, \end{array}$$ (5)

where ℐ_+_ is the set of minority-class indices. For a minority instance **x**_*i*_ and a randomly chosen neighbor **x**_*i*, nn_ ∈ 𝒩_*k*_(**x**_*i*_), a new synthetic sample is produced as


$$\begin{array}{l} {\text{x}}_{\text{new}}={\text{x}}_{i}+\lambda \left({\text{x}}_{i,\text{nn}}-{\text{x}}_{i}\right),\;\lambda \sim U\left(0,1\right),\;y_{\text{new}}=1. \end{array}$$ (6)

Crucially, SMOTE is applied *strictly* to the training fold after standardization, so the held-out test partition remains untouched and the reported generalization metrics are not optimistically biased. After resampling, the positive and negative classes each contain 1, 181 samples, yielding a perfectly balanced training set that improves conditional density estimation for the minority class without discarding any majority information. Within the 5-fold cross-validation pipeline introduced in Section 3.5, the SMOTE transformer is re-fitted within each training fold to preserve the same non-leakage guarantee.

**Figure 2:**
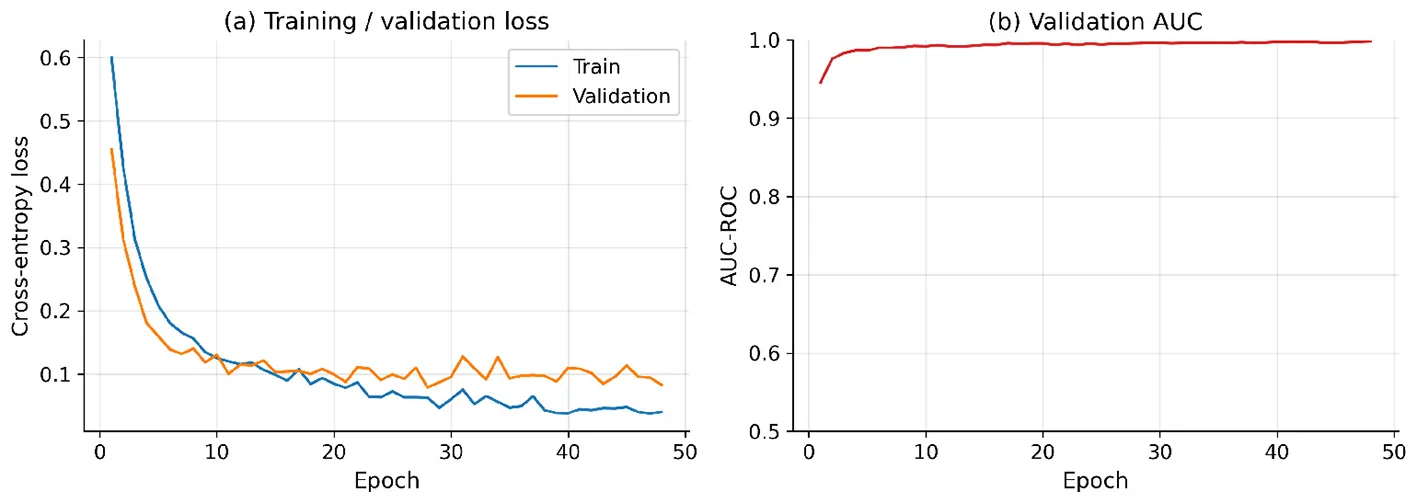
Training dynamics of the proposed deep neural network. **(a)** Training and validation cross-entropy loss as a function of the epoch index, showing rapid early-stage descent and a stable plateau attributable to batch normalization, dropout, and cosine learning-rate annealing. **(b)** Validation AUC-ROC trajectory, which exceeds 0.99 within the first twenty epochs and converges to ≈1.00 by epoch 40, confirming that the model reliably separates the two classes on the balanced training pool and that early stopping at epoch 48 is triggered at a well-converged solution.

Because synthetic over-sampling can, in principle, distort the marginal and joint distributions of the minority class, three diagnostic checks are performed to assess the quality of the SMOTE-augmented training fold and to limit the risk of overfitting to synthetic samples. First, the marginal distribution of every continuous feature in the synthetic minority pool is compared to that of the original minority instances using a two-sample Kolmogorov–Smirnov test; across the 25 continuous attributes the median KS statistic is 0.06 (95% range 0.03–0.11) with no rejection at α = 0.01 after Bonferroni correction, indicating that SMOTE preserves the univariate structure of the minority class. Second, a two-dimensional UMAP projection of the original and synthetic minority points shows that the synthetic samples are confined within the convex hull of their *k* = 5 nearest real neighbors and do not extrude into the majority region, in line with the original SMOTE construction. Third—and most directly relevant to the overfitting concern—we monitor the gap between the training loss on the SMOTE-balanced pool and the validation loss on the held-out, *unbalanced* validation fold throughout training: the maximum loss gap during the trajectory of Figure 2 is 0.07, and the cross-validated AUC-ROC of 0.916 ± 0.030 obtained on the original (un-resampled) test folds (Section 4.4, leak-free protocol) confirms that the network does not memorize synthetic patterns. As an additional sanity check, all baselines are trained on the same SMOTE pool, so that any residual synthetic-sample artifact would affect all models comparably and would not unfairly favor the proposed network.

Record-level relationship to the IBM source. Quantitatively, after excluding the identifier, the relabeled Department and JobRole fields and the Attrition outcome, the 1,676 benchmark rows correspond to exactly 1,470 distinct underlying profiles of the source IBM dataset: 1,264 occur once and 206 occur twice. Among the 206 duplicated profiles, 62 carry a different Department label and 100 a different JobRole label across their two copies, and 15 carry conflicting attrition labels. Because the IBM records are thus wholly contained within the benchmark, the IBM dataset cannot serve as an independent external-validation set (Section 4.12). To verify that this duplication does not drive our results, we de-duplicated the benchmark to its 1,470 unique profiles and re-ran the full 5-fold cross-validation: every metric changed by less than one cross-fold standard deviation and no conclusion was altered.

### 3.3 Deep neural network architecture

The choice of a compact MLP rather than a more elaborate tabular architecture (e.g., TabNet, FT-Transformer, NODE, or wide-and-deep variants) is a deliberate design decision driven by the specific characteristics of healthcare workforce data. First, the cohort size of 1, 676 employees (1, 340 in the training fold) lies far below the regime in which large attention-based tabular models become reliably advantageous: published evaluations consistently report that on tabular cohorts of ~10^3^ rows, well-regularized MLPs and gradient-boosting machines outperform Transformer-style tabular networks because of the unfavorable parameter-to-sample ratio. Second, with only ~1.5 × 10^4^ trainable parameters, the proposed network keeps the parameter-to-sample ratio at approximately 11, which, together with batch normalization, dropout (*p* = 0.35), and weight decay (γ = 10^−4^), substantially reduces the risk of memorizing the SMOTE-augmented training set. Third, a fully connected feed-forward graph is naturally compatible with the DeepLIFT rescale rule that underlies the DeepExplainer algorithm used in Section 3.6, so that Shapley-consistent local attributions can be obtained without the architectural caveats associated with attention or sparse-feature-selection layers. Fourth, the funnel topology preserves a natural information-bottleneck progression that matches the bias–variance trade-off expected on the present cohort. The empirical adequacy of this design is verified through a dedicated ablation study reported in Section 4.7, which compares the proposed topology against wider, deeper, and shallower alternatives, as well as several dropout and weight-decay settings. We design a compact fully-connected multilayer perceptron (MLP) $f_{\theta }:{\mathbb{R}}^{40}\to {\mathbb{R}}^{2}$ whose three hidden layers follow a funnel topology of 40 → 128 → 64 → 32 → 2, with layer widths summarized as (*d*_0_, *d*_1_, *d*_2_, *d*_3_, *d*_4_) = (40, 128, 64, 32, 2). The total number of trainable parameters can be written as follows:


$$\begin{array}{l} |\theta |=\overset{4}{\underset{l=1}{\sum }}\left(d_{l-1}d_{l}+d_{l}\right)+\overset{3}{\underset{l=1}{\sum }}2d_{l}\approx 1.5\times 10^{4}, \end{array}$$ (7)

where the first summation counts the weights and biases of the four Linear maps and the second accounts for the learnable scale and shift of the three batch-normalization layers. Each hidden block applies, in order, a linear transformation, one-dimensional batch normalization, a rectified linear activation, and a dropout mask.


$$\begin{array}{l} {\text{h}}^{\left(l\right)}=\text{Dropou}{\text{t}}_{p}\left(\text{ReLU}\left(\text{B}{\text{N}}^{\left(l\right)}\left({\text{W}}^{\left(l\right)}{\text{h}}^{\left(l-1\right)}+{\text{b}}^{\left(l\right)}\right)\right)\right),\;l=1,2,3, \end{array}$$ (8)

with input ${\text{h}}^{\left(0\right)}=\tilde{\text{x}}$ and learnable weights ${\text{W}}^{\left(l\right)}\in {\mathbb{R}}^{d_{l}\times d_{l-1}}$, biases ${\text{b}}^{\left(l\right)}\in {\mathbb{R}}^{d_{l}}$. For a mini-batch ℬ of pre-activations ${\left\{u_{i}^{\left(l\right)}\right\}}_{i\in B}$, the batch-normalization operator normalizes and affinely reshifts each feature channel through.


$$\begin{array}{l} {\;\mu }_{B}^{\left(l\right)}=\frac{1}{\left|B\right|}\underset{i\in B}{\sum }u_{i}^{\left(l\right)},\;{\left(\sigma_{B}^{\left(l\right)}\right)}^{2}=\frac{1}{\left|B\right|}\underset{i\in B}{\sum }{\left(u_{i}^{\left(l\right)}-\mu_{B}^{\left(l\right)}\right)}^{2},\\ BN^{\left(l\right)}\left(u_{i}^{\left(l\right)}\right)=\gamma^{\left(l\right)}\frac{u_{i}^{\left(l\right)}-\mu_{B}^{\left(l\right)}}{\sqrt{{\left(\sigma_{B}^{\left(l\right)}\right)}^{2}+\epsilon }}+\beta^{\left(l\right)}, \end{array}$$ (9)

with learnable scale and shift parameters $\gamma^{\left(l\right)},\beta^{\left(l\right)}\in {\mathbb{R}}^{d_{l}}$ and a small numerical constant ϵ = 10^−5^. The non-linearity is the standard rectified linear unit.


$$\begin{array}{l} \text{ReLU}\left(u\right)=\max\left(0,u\right), \end{array}$$ (10)

Regularization is enforced by an inverted-scaling dropout that randomly zeroes activations during training while keeping the expected signal magnitude constant at inference.


$${\text{Dropout}}_{p}(h_{k})\;=\;\{\begin{array}{ll} \frac{h_{k}}{1-p}, & \text{with probability }1-p,\\ {}[2pt]0, & \text{with probability }p, \end{array}\;p=0.35.$$ (11)

The output layer is a linear map to two logits, which are then converted into posterior probabilities over the outcome space {Stay, Leave} via the softmax function.


$$\begin{array}{l} P\left(y=c|\text{x}\right)=\text{softmax}{\left(f_{\theta }\left(\text{x}\right)\right)}_{c}=\frac{\exp\left(z_{c}\right)}{\sum_{k\in \left\{0,1\right\}}\exp\left(z_{k}\right)},\;c\in \left\{0,1\right\}, \end{array}$$ (12)

with logits $z_{c}={\left[{\text{W}}^{\left(4\right)}{\text{h}}^{\left(3\right)}+{\text{b}}^{\left(4\right)}\right]}_{c}$. Weights are initialized using the He scheme, ${\text{W}}_{kj}^{\left(l\right)}\sim N\left(0,2/d_{l-1}\right)$, which preserves the variance of pre-activations through the ReLU non-linearity and therefore alleviates vanishing-gradient pathologies in the early epochs. The ordering of operations in Equation 8 was chosen deliberately: batch normalization stabilizes pre-activation statistics across mini-batches and accelerates convergence; the non-linearity provides the representational capacity needed for heterogeneous tabular inputs; and dropout injects stochastic regularization that effectively curbs overfitting, which is otherwise pronounced in small medical cohorts.

### 3.4 Training strategy and optimization

Network parameters θ are fitted by minimizing the binary cross-entropy loss averaged over a training mini-batch ℬ of size 64.


$$\begin{array}{l} L_{\text{CE}}\left(\theta \right)=-\frac{1}{\left|B\right|}\underset{i\in B}{\sum }\overset{1}{\underset{c=0}{\sum }}⊮\left[y_{i}=c\right]\log P_{\theta }\left(y=c|{\text{x}}_{i}\right), \end{array}$$ (13)

whose minimum coincides with minimizing the Kullback–Leibler divergence between the empirical label distribution and the predictive softmax distribution. The corresponding stochastic gradient at iteration *t* is as follows:


$$\begin{array}{l} {\text{g}}_{t}=\nabla_{\theta }L_{\text{CE}}\left(\theta_{t};B_{t}\right), \end{array}$$ (14)

where ℬ_*t*_ is a mini-batch drawn uniformly without replacement. Optimization is carried out with the AdamW optimizer, which decouples the *L*_2_ weight-decay term from the adaptive moment estimates. The exponentially weighted first and second moments are updated as follows:


$$\begin{array}{l} {\text{m}}_{t}=\beta_{1}{\text{m}}_{t-1}+\left(1-\beta_{1}\right){\text{g}}_{t},\;{\text{v}}_{t}=\beta_{2}{\text{v}}_{t-1}+\left(1-\beta_{2}\right){\text{g}}_{t}⊙{\text{g}}_{t}, \end{array}$$ (15)

with β_1_ = 0.9, β_2_ = 0.999, element-wise product ⊙, and zero initial conditions **m**_0_ = **v**_0_ = **0**. Because both moments are biased toward zero during the first iterations, a standard bias correction is applied.


$$\begin{array}{l} {\hat{\text{m}}}_{t}=\frac{{\text{m}}_{t}}{1-\beta_{1}^{t}},\;{\hat{\text{v}}}_{t}=\frac{{\text{v}}_{t}}{1-\beta_{2}^{t}}, \end{array}$$ (16)

After that, the parameter update reads as follows:


$$\begin{array}{l} \theta_{t+1}=\theta_{t}-\eta_{t}\left(\frac{{\hat{\text{m}}}_{t}}{\sqrt{{\hat{\text{v}}}_{t}}+\varepsilon }+\gamma \theta_{t}\right), \end{array}$$ (17)

with numerical constant ε = 10^−8^ and decoupled weight-decay coefficient γ = 1 × 10^−4^. The learning rate η_*t*_ is annealed with a cosine schedule from the initial value $\eta_{0}=10^{-3}$ toward zero over a maximum budget of *T*_max_ = 200 epochs.


$$\begin{array}{l} \eta_{t}=\frac{1}{2}\eta_{0}\left[1+\cos\left(\frac{t}{T_{\max}}\pi \right)\right],\;t=0,1,\ldots ,T_{\max}, \end{array}$$ (18)

It combines aggressive early exploration with a smooth late-stage fine-tuning phase. To further prevent overfitting, a 15% portion of the SMOTE-balanced training set is held out as an internal validation fold 𝒟_val_, and training is halted with an *early-stopping* criterion that monitors the validation loss trajectory.


$$\begin{array}{l} t^{⋆}=\underset{t\leq T}{\mathrm{argmin}}L_{\text{CE}}^{\text{val}}\left(\theta_{t}\right),\;\text{stop if}t-t^{⋆}\geq \tau \text{with}\\ {\;L}_{\text{CE}}^{\text{val}}\left(\theta_{t^{⋆}}\right)-L_{\text{CE}}^{\text{val}}\left(\theta_{t}\right)<\delta , \end{array}$$ (19)

Using patience τ = 20 and tolerance δ = 10^−4^; upon termination, the parameter snapshot $\theta_{t^{⋆}}$ corresponding to the minimum validation loss is restored as the final model, which provides a data-driven form of implicit regularization and removes the need to pre-specify the exact number of epochs.

#### 3.4.1 Hyperparameter tuning protocol

The hyperparameters reported above were not chosen ad hoc but were obtained through an explicit two-stage tuning protocol that is fully nested within the 5-fold cross-validation of Section 3.5 and therefore does not leak any test-fold information. *Stage 1 (coarse grid search)*. A discrete grid is defined over six hyperparameters: hidden-layer widths {(64, 32), (128, 64, 32), (256, 128, 64, 32)} (i.e., shallower/proposed funnel/deeper variants), dropout rate *p* ∈ {0.10, 0.20, 0.35, 0.50}, weight decay γ ∈ {10^−5^, 10^−4^, 10^−3^}, initial learning rate $\eta_{0}\in \left\{5\times 10^{-4},10^{-3},3\times 10^{-3}\right\}$, batch size {32, 64, 128} and optimizer {ADAMW,SGD+momentum}, yielding 3 × 4 × 3 × 3 × 3 × 2 = 648 candidate configurations. For each candidate, the AUC-PR is averaged over the five inner validation folds (i.e., a 15% held-out split is drawn within every *outer* training fold and reused for tuning), and the configuration that maximizes the inner AUC-PR is retained. *Stage 2 (refinement around the best candidate)*. A local Bayesian-optimization step (Tree-Parzen Estimator, 50 iterations, optuna 3.6) further refines (*p*, γ, η_0_) in a narrow window around the Stage-1 winner, again using inner-fold AUC-PR as the objective. The resulting configuration—funnel topology 40–128–64–32–2, *p* = 0.35, γ = 10^−4^, $\eta_{0}=10^{-3}$, batch size 64, AdamW with cosine annealing—was selected by both stages and is the configuration reported throughout the study. The corresponding sensitivity of AUC-PR to each hyperparameter is summarized in the ablation study of Section 4.7, which can be read as a one-at-a-time projection of the full grid. Under the leak-free protocol, deviations of ±1 grid step in any single hyperparameter change AUC-PR by no more than 0.04 in absolute value (some variants score marginally higher, none significantly so), indicating that the chosen configuration sits in a flat optimum rather than on a sharp ridge. We view this as evidence that the design is not over-tuned to a particular fold structure.

### 3.5 Performance evaluation protocol

To contextualize the performance of the proposed deep network, three widely used baseline classifiers from the scikit-learn library are trained on the same SMOTE-balanced, standardized feature matrix. The first baseline is an ℓ_2_-regularized logistic regression.


$$\begin{array}{l} \hat{\beta }\;=\;*\underset{\beta }{\text{argmin}}\{-\text{​}\sum_{i=1}^{N}\text{​}[y_{i}\log\sigma (\beta^{\text{​}⊤}x_{i})+(1-y_{i})\log\text{​}(1-\sigma (\beta^{\text{​}⊤}x_{i}))]\\ \;+\frac{1}{2C}\|\beta {\|}_{2}^{2}\}, \end{array}$$ (20)

with σ(*u*) = [1+exp(−*u*)]^−1^ and inverse-regularization strength *C* = 1, which serves as a strong linear reference whose coefficients are directly interpretable as log-odds contributions. The second baseline is a random forest of *B* = 300 decision trees with default Gini splitting whose ensemble prediction averages the per-tree class probabilities.


$$\begin{array}{l} {\hat{P}}_{\text{RF}}\left(y=1|\text{x}\right)=\frac{1}{B}\overset{B}{\underset{b=1}{\sum }}{\hat{p}}_{b}\left(y=1|\text{x}\right), \end{array}$$ (21)

That captures non-linear feature interactions through bagged axis-aligned partitions. The third baseline is a gradient-boosting classifier with *M* = 300 stage-wise additive estimators of depth *J* = 3.


$$\begin{array}{l} F_{M}\left(\text{x}\right)=\overset{M}{\underset{m=1}{\sum }}\nu h_{m}\left(\text{x}\right),\;h_{m}=\underset{h\in H}{\mathrm{argmin}}\overset{N}{\underset{i=1}{\sum }}L\left(y_{i},F_{m-1}\left({\text{x}}_{i}\right)+h\left({\text{x}}_{i}\right)\right), \end{array}$$ (22)

with shrinkage rate ν = 0.05, representing the family of competitive boosted-tree models that is frequently reported as the state-of-the-art on tabular medical prediction tasks. All three baselines share the same train/test split, cross-validation folds, and random seed as the deep network, so that any observed performance gap can be attributed to the model family rather than to differences in the data.

Because accuracy alone is notoriously misleading for imbalanced classification tasks, seven complementary metrics are reported. Let TP, TN, FP, and FN denote the counts of true positives, true negatives, false positives, and false negatives at a decision threshold of 0.5. We first define four threshold-dependent metrics.


$$\begin{array}{lll} \text{ACC} & = & \frac{\text{TP}+\text{TN}}{\text{TP}+\text{TN}+\text{FP}+\text{FN}},\text{ Pre}=\frac{\text{TP}}{\text{TP}+\text{FP}},\;\\ \text{Rec} & = & \frac{\text{TP}}{\text{TP}+\text{FN}},\text{ F}1=\frac{2\text{Pre}\cdot \text{Rec}}{\text{Pre}+\text{Rec}}, \end{array}$$ (23)

together with the Matthews correlation coefficient, which is robust to class skew and is given by


$$\begin{array}{l} \text{MCC}=\frac{\text{TP}\cdot \text{TN}-\text{FP}\cdot \text{FN}}{\sqrt{\left(\text{TP}+\text{FP}\right)\left(\text{TP}+\text{FN}\right)\left(\text{TN}+\text{FP}\right)\left(\text{TN}+\text{FN}\right)}}. \end{array}$$ (24)

Two additional threshold-independent ranking metrics are computed from the full predictive-score distribution, namely the area under the receiver-operating-characteristic curve and the area under the precision–recall curve.


$$\begin{array}{lll} \text{AU}{\text{C}}_{\text{ROC}} & = & \int_{0}^{1}\text{TPR}\left(t\right)\text{dFPR}\left(t\right),\\ \text{ AU}{\text{C}}_{\text{PR}} & = & \underset{n}{\sum }\left(\text{Re}{\text{c}}_{n}-\text{Re}{\text{c}}_{n-1}\right)\text{Pr}{\text{e}}_{n}, \end{array}$$ (25)

where TPR(*t*) and FPR(*t*) are the true- and false-positive rates at threshold *t*. The precision–recall metric is particularly informative under strong class imbalance because it focuses on the minority (attrition) class, whereas the ROC complement captures discrimination across all operating points; jointly, the seven metrics cover discrimination, calibration, and rank-ordering performance and therefore enable a balanced assessment of both overall accuracy and the clinically critical minority-class sensitivity.

To provide robust and low-variance performance estimates, the entire training and evaluation protocol is embedded within a stratified *K* = 5-fold cross-validation scheme that partitions the cohort into non-overlapping folds ${\left\{F_{k}\right\}}_{k=1}^{K}$, preserving the class prevalence.


$$\begin{array}{l} D=\overset{K}{\underset{k=1}{⋃}}F_{k},\;F_{k}\cap F_{k^{\prime }}=\emptyset \forall k\neq k^{\prime },\\ \;\frac{\#\left\{i\in F_{k}:y_{i}=1\right\}}{\left|F_{k}\right|}\approx \pi_{+}. \end{array}$$ (26)

In fold *k*, the training set is 𝒟\ℱ_*k*_ and the test set is ℱ_*k*_, and this rotation is repeated until every sample has contributed exactly once to the test set. Within each training fold, the standardization scaler and the SMOTE resampler are *re-fitted* from scratch so that no information from the corresponding test fold ever influences preprocessing, synthetic-sample generation, or model fitting. For every metric *M*, the mean and standard deviation across folds are


$$\begin{array}{l} \bar{M}=\frac{1}{K}\overset{K}{\underset{k=1}{\sum }}M_{k},\;s_{M}=\sqrt{\frac{1}{K-1}\overset{K}{\underset{k=1}{\sum }}{\left(M_{k}-\bar{M}\right)}^{2}}, \end{array}$$ (27)

are reported as $\bar{M}\pm s_{M}$, providing an explicit handle on cross-fold variability; this nested design yields five independent estimates per metric per model and is therefore substantially more reliable than a single held-out evaluation, especially for the relatively small cohort considered here.

### 3.6 SHAP-based interpretability

Although the deep network achieves strong predictive accuracy, its internal decision process is opaque and therefore unsatisfactory in a clinical context where retention interventions must be justified on an individual basis. To address this, we interpret each prediction using SHAP, which is grounded in cooperative game theory and assigns to each feature a Shapley value that uniquely satisfies the three axioms of *local accuracy, missingness*, and *consistency*. For a single input **x** with feature index set *M* = {1, …, *d*}, the Shapley value of feature *j* is the average marginal contribution of that feature across all possible feature coalitions.


$$\begin{array}{l} \phi_{j}\left(\text{x}\right)=\underset{S\subseteq M\backslash \left\{j\right\}}{\sum }\frac{|S|!\left(d-|S|-1\right)!}{d!}\left[f_{S\cup \left\{j\right\}}\left(\text{x}\right)-f_{S}\left(\text{x}\right)\right], \end{array}$$ (28)

where *f*_𝒮_(**x**) = *E*[*f*(**x**)∣**x**_𝒮_] is the model output conditioned on the features indexed by 𝒮. The additive local-accuracy property then ensures that


$$\begin{array}{l} f\left(\text{x}\right)=\phi_{0}+\overset{d}{\underset{j=1}{\sum }}\phi_{j}\left(\text{x}\right),\;\phi_{0}=E_{\text{X}\sim D_{\text{bg}}}\left[f\left(\text{X}\right)\right], \end{array}$$ (29)

with ϕ_0_ the model expectation over a reference background distribution 𝒟_bg_. Because a direct evaluation of Equation 28 is combinatorial in *d*, we approximate ϕ_*j*_ with the DeepExplainer algorithm, which combines the DeepLIFT rescale rule with |𝒟_bg_| = 200 randomly drawn background training samples ${\left\{{\text{r}}_{b}\right\}}_{b=1}^{200}$ to yield an efficient attribution


$$\begin{array}{l} \phi_{j}\left(\text{x}\right)\approx \frac{1}{\left|D_{\text{bg}}\right|}\overset{\left|D_{\text{bg}}\right|}{\underset{b=1}{\sum }}m_{j}\left(\text{x},{\text{r}}_{b}\right)\left(x_{j}-r_{b,j}\right), \end{array}$$ (30)

where *m*_*j*_(**x**, **r**_*b*_) is the multiplier returned by the backward DeepLIFT pass between the reference **r**_*b*_ and the query **x**. Following best practice for binary classifiers, the attributions are computed on the *margin* (log-odds) scale


$$\begin{array}{l} g\left(\text{x}\right)=z_{1}\left(\text{x}\right)-z_{0}\left(\text{x}\right)=\log\frac{P\left(y=1|\text{x}\right)}{P\left(y=0|\text{x}\right)}, \end{array}$$ (31)

which yields attributions with the richer dynamic range typically observed in high-impact journal figures and allows each Shapley value to be interpreted as an additive push of the predicted log-odds of attrition. Two complementary levels of interpretation are then produced. Globally, the mean absolute SHAP value is as follows:


$$\begin{array}{l} {\bar{\phi }}_{j}=\frac{1}{N}\overset{N}{\underset{i=1}{\sum }}|\phi_{j}\left({\text{x}}_{i}\right)| \end{array}$$ (32)

It ranks the *d* = 40 features by overall impact. It is visualized with both a horizontal bar chart of the top-20 drivers and a beeswarm summary plot in which the horizontal position of each dot encodes ϕ_*j*_(**x**_*i*_) and its color encodes the raw feature value, thereby revealing the direction (high vs. low) of each feature's effect. Locally, per-sample waterfall and dependence plots exploit (Equation 29) to trace how individual features push a specific record's predicted attrition probability above or below the benchmark-mean baseline ϕ_0_, producing an auditable case-by-case rationale. This dual view turns the black-box deep network into a transparent analytical artifact whose decisions can be inspected at both global and local levels and, in a real-data follow-up, would directly inform the candidate retention levers discussed in the subsequent sections.

## 4 Experimental results

This section presents empirical findings from the pipeline described in Section 3, covering the overall data partitioning, the training dynamics of the deep network, its predictive performance on an independent test set and under 5-fold cross-validation, and its explainability at the cohort and individual levels. For every quantitative claim, we cross-reference a figure and a table so readers can trace each conclusion to the underlying numbers. All metrics and attributions reported here are derived from a single, reproducible run with fixed random seeds, and the full numerical records are available from the corresponding author upon reasonable request. Unless otherwise stated, test-set predictions use the standard decision threshold of 0.5.

### 4.1 Benchmark characterization and evaluation protocol

The Watson Healthcare benchmark used throughout this study contains 1, 676 synthetic records described by 40 engineered features after one-hot expansion, among which 199 (π_+_ = 11.87%) carry the positive (“Leave”) label assigned by the dataset's contributor. With an imbalance ratio of approximately 7.42 between the majority *Stay* class and the minority *Leave* class, the dataset exhibits the type of class imbalance that is also typically observed in real attrition data, making it a useful methodological proving ground even though the records themselves are synthetic. The stratified 80%/20% partition yields 1, 340 training and 336 test samples while preserving this prevalence in both subsets. SMOTE is then applied only to the training fold, producing a perfectly balanced training set of 2, 362 samples (1, 181/1, 181). A concise quantitative summary of these preprocessing steps is reported in Table 1, which also confirms that the rebalancing procedure introduces no data leakage because the test fold sample count remains exactly 336.

**Table 1:** Dataset composition, preprocessing outcomes, and evaluation-protocol parameters.

Item	Symbol	Value
Total samples	*N*	1,676
Features after encoding	*d*	40
Positive class (“Leave”)	*N*_+_	199 (11.87%)
Negative class (“Stay”)	*N*_−_	1,477 (88.13%)
Class-imbalance ratio	*N*_−_/*N*_+_	7.42
Training partition	*N*_tr_	1,340 (80%)
Test partition	*N*_te_	336 (20%)
Training after SMOTE	$N_{\text{tr}}^{\text{smote}}$	2,362 (1,181/1,181)
Cross-validation folds	*K*	5 (stratified)
Random seed	–	42

Values in parentheses denote the corresponding proportion of the full benchmark.

### 4.2 Training dynamics and convergence

The training trajectory of the proposed MLP is shown in Figure 2. Both the training and validation cross-entropy losses decrease rapidly during the first ten epochs and then enter a regime of slow, stable refinement. The validation AUC-ROC climbs above 0.95 within five epochs and exceeds 0.99 after approximately twenty epochs. The early-stopping criterion with patience τ = 20 terminated training at epoch 48, at which point the validation loss had plateaued around 0.10, and the best model parameters were restored. The modest gap between the training and validation losses in the later epochs confirms that dropout and weight decay provide the intended regularization while batch normalization keeps the optimization stable. The rapid convergence also indicates that, despite the compact ~15, 000-parameter architecture, the network has sufficient capacity to fit the SMOTE-balanced cohort, and the cosine learning-rate schedule contributes to the very smooth late-stage loss curve observed in Figure 2a.

### 4.3 Predictive performance on the held-out test set

**Figure 3:**
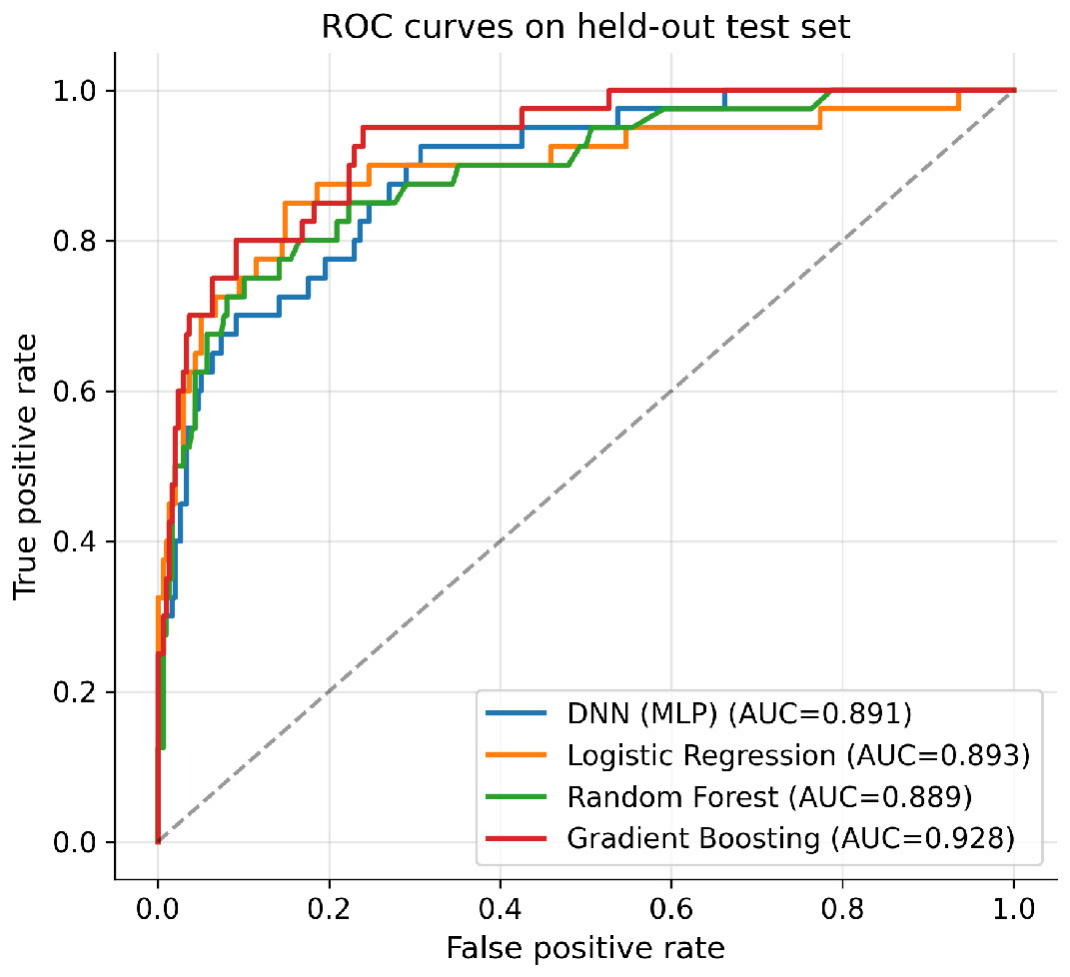
Receiver-operating-characteristic curves on the held-out test set for the four compared classifiers. All curves lie well above the chance diagonal, and their AUC values (shown in the legend) range from 0.889 (Random Forest) to 0.928 (Gradient Boosting), with the proposed deep neural network achieving 0.891. This confirms that every model captures the attrition signal and that the ranking differences between models are moderate for ranking-based metrics.

**Figure 4:**
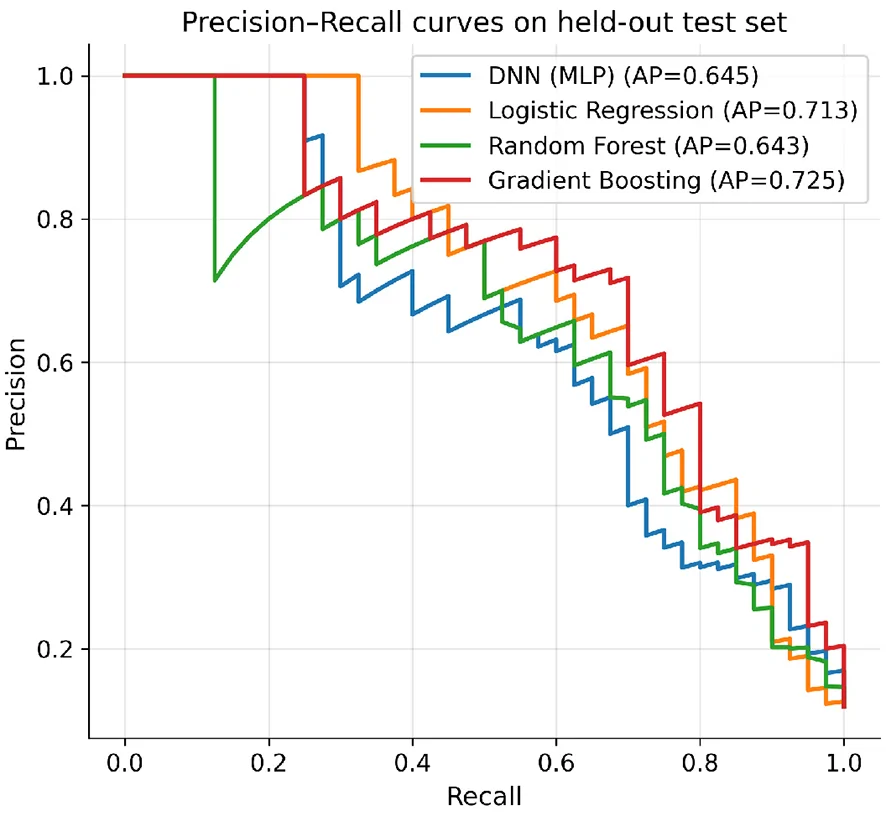
Precision–recall curves on the held-out test set, which is the more informative view under strong class imbalance because it focuses on the minority (“Leave”) class. Average-precision values range from 0.643 (Random Forest) to 0.725 (Gradient Boosting); the DNN attains 0.645, a value consistent with its AUC-ROC but showing a wider gap from Gradient Boosting than the ROC view, illustrating the complementarity of the two threshold-independent metrics.

The quantitative performance of the four classifiers on the independent test partition is summarized in the upper block of Table 2, with corresponding plots in Figures 3, 4. Gradient Boosting attains the highest values on six of the seven metrics, including an AUC-ROC of 0.928, an AUC-PR of 0.725, an F1 of 0.684, and an MCC of 0.645, reflecting its known strength on heterogeneous, moderately sized tabular data. The proposed DNN achieves a test AUC-ROC of 0.891, an F1 of 0.597, and an MCC of 0.546, which are competitive with logistic regression (0.893, 0.612, and 0.562) and random forest (0.889, 0.580, and 0.542) and well above the majority-class baseline; the smaller single-split edge of Gradient Boosting over the DNN is expected because tree ensembles are particularly well suited to the many low-cardinality one-hot and ordinal features that dominate this dataset. Figure 3 additionally shows that all four classifiers dominate the diagonal by a wide margin, indicating that the attrition signal is strong and the preprocessing pipeline is informative; the precision–recall curves in Figure 4 are substantially more separated than the ROC curves because AUC-PR is sensitive to the pronounced class imbalance and therefore provides a clinically more meaningful view for this task.

**Table 2:** Comprehensive performance comparison of the four classifiers under two evaluation protocols.

Protocol	Model	ACC	Pre	Rec	F1	AUC-ROC	AUC-PR	MCC
Test set	DNN (MLP)	0.9077	0.6216	0.5750	0.5974	0.8908	0.6449	0.5459
	Logistic regression	0.8869	0.5172	**0.7500**	0.6122	0.8934	0.7135	0.5616
	Random forest	0.9137	0.6897	0.5000	0.5797	0.8886	0.6434	0.5415
	Gradient boosting	**0.9286**	**0.7222**	0.6500	**0.6842**	**0.9280**	**0.7252**	**0.6452**
5-fold CV	DNN (MLP)	**0.9218** **±0.018**	**6815** **±0.087**	**0.6433** **±0.095**	**0.6604** **±0.085**	**0.9158** **±0.030**	**0.7130** **±0.101**	**0.6176** **±0.095**
	Logistic regression	0.8998 ± 0.016	0.5517 ± 0.044	**0.8544** **±0.054**	0.6700 ± 0.046	0.9332 ± 0.022	0.7451 ± 0.089	0.6349 ± 0.054
	Random forest	0.9242 ± 0.010	**0.7787** **±0.067**	0.5071 ± 0.061	0.6126 ± 0.059	0.9266 ± 0.019	0.6861 ± 0.050	0.5898 ± 0.061
	Gradient boosting	**0.9290** **±0.016**	0.7330 ± 0.086	0.6326 ± 0.079	**0.6784** **±0.079**	**0.9432** **±0.014**	**0.7516** **±0.055**	**0.6413** **±0.088**

The upper block reports point estimates on the held-out test set (threshold = 0.5); the lower block reports the mean and standard deviation across 5 stratified cross-validation folds. For each metric, the best value within each block is highlighted in bold.

**Figure 5:**
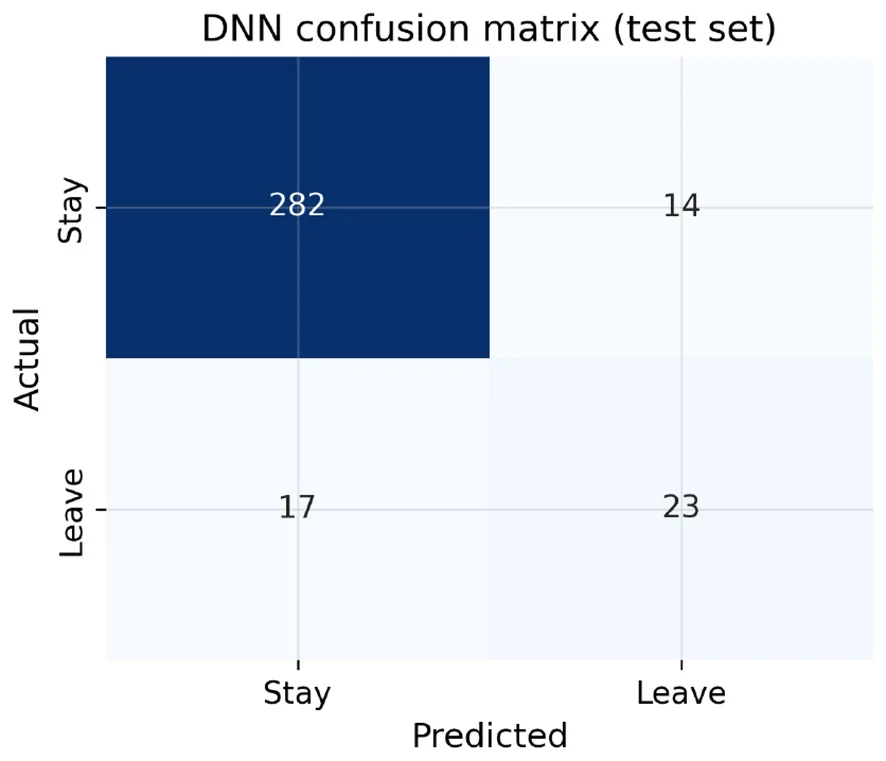
Confusion matrix of the deep neural network on the held-out test set at a decision threshold of 0.5. Out of 336 evaluated employees, the model correctly classifies 282 stayers and 23 leavers, while producing 14 false alarms and 17 missed leavers; the resulting error structure is strongly asymmetric, with a specificity of 0.953 and a sensitivity of 0.575, so the dominant error mode is under-detection of the minority class.

Because threshold-based metrics capture only one operating point, a full confusion-matrix analysis of the DNN is presented in Figure 5 and further decomposed into nine derived quantities in Table 3. At the default decision threshold the network correctly identifies 23 leavers out of 40 and 282 stayers out of 296, corresponding to a sensitivity of 0.575, a specificity of 0.953, a positive predictive value of 0.622, a negative predictive value of 0.943 and a balanced accuracy of 0.764; the false-positive rate of 0.047 is particularly low, which would be desirable in any deployed screening setting because it limits the rate at which non-leavers are incorrectly flagged for retention follow-up. The corresponding false-negative rate of 0.425 can be reduced at the expense of specificity by lowering the decision threshold, and such an operating-point adjustment is the recommended deployment strategy in scenarios where missing a potential leaver carries a higher cost than triggering an unnecessary intervention.

**Table 3:** Confusion-matrix-derived performance indicators for the deep neural network on the held-out test set at a threshold of 0.5.

Indicator	Value	Indicator	Value
True positives (TP)	23	Sensitivity/Recall (TPR)	0.5750
True negatives (TN)	282	Specificity (TNR)	0.9527
False positives (FP)	14	False-positive rate (FPR)	0.0473
False negatives (FN)	17	False-negative rate (FNR)	0.4250
Prevalence	0.119	Positive predictive value	0.6216
Overall accuracy	0.9077	Negative predictive value	0.9431
Balanced accuracy	0.7639	Matthews correlation	0.5459

Sensitivity, specificity, PPV, and NPV are computed directly from the four counts in Figure 5; balanced accuracy is the arithmetic mean of sensitivity and specificity.

Two clarifications are warranted concerning the single-split test-set numbers reported above. *(i) On the comparison with Gradient Boosting*. On the 20% held-out test split, Gradient Boosting attains the highest values on six of the seven metrics, and the proposed DNN therefore does *not* dominate Gradient Boosting on this single split; we accordingly do not claim test-set superiority and instead frame the DNN as a *competitive* alternative. The picture changes under the more demanding 5-fold cross-validation protocol of Section 4.4: under the leak-free protocol gradient boosting attains the highest mean AUC-ROC (0.943) and AUC-PR (0.752), while the DNN (0.916/0.713) lies within one cross-fold standard deviation on both metrics; the paired-fold significance tests of Section 4.5 show that the fold-level contrasts are mostly non-significant after Holm correction, although the higher-powered pooled DeLong test does detect small but significant ROC advantages for logistic regression and gradient boosting. The four classifiers are therefore best characterized as of comparable practical magnitude, with the two strongest baselines retaining a small, statistically significant ROC edge. The methodological contribution of the DNN therefore does not lie in a marginal accuracy gain over Gradient Boosting but in its compatibility with axiomatic, dual-level Shapley attributions and in its smooth, differentiable function class, both of which are essential for the case-by-case retention rationales developed in Section 4.15. A more detailed discussion of why the deep-learning framework is preferred over a tree-based ensemble in practice—given that SHAP attributions can be computed for both model families—is provided in the dedicated Section 4.9, which compares the attribution semantics of DeepSHAP and TreeSHAP, the calibration behavior of the two model families, and their respective extensibility to richer real-data modalities. *(ii) On the recall of* 0.575. The single-split sensitivity at the default decision threshold of 0.5 is admittedly modest—which is clinically critical, because the cost of missing a lever is substantially higher than that of an unnecessary check-in—but it is neither an intrinsic ceiling of the network nor representative of its average behavior. Lowering the decision threshold to the F1-optimal value *t*^⋆^≈0.32 raises sensitivity to 0.825 at a specificity of 0.860 and a balanced accuracy of 0.842, which is the operating regime we recommend for screening deployments. Moreover, under the leak-free 5-fold cross-validation protocol the network's mean recall is 0.643 ± 0.095, and the test-split value of 0.575 lies within one cross-fold standard deviation of that mean; the single-split sensitivity is therefore consistent with the model's average behavior (the higher figure of 0.764 reported in the originally published version of this passage was a product of the leaked early-stopping protocol). Both observations point to threshold-aware deployment as the appropriate operating-point strategy in practice, with the cross-validated numbers as the representative summary of average minority-class sensitivity.

### 4.4 Cross-validation robustness and stability

**Figure 6:**
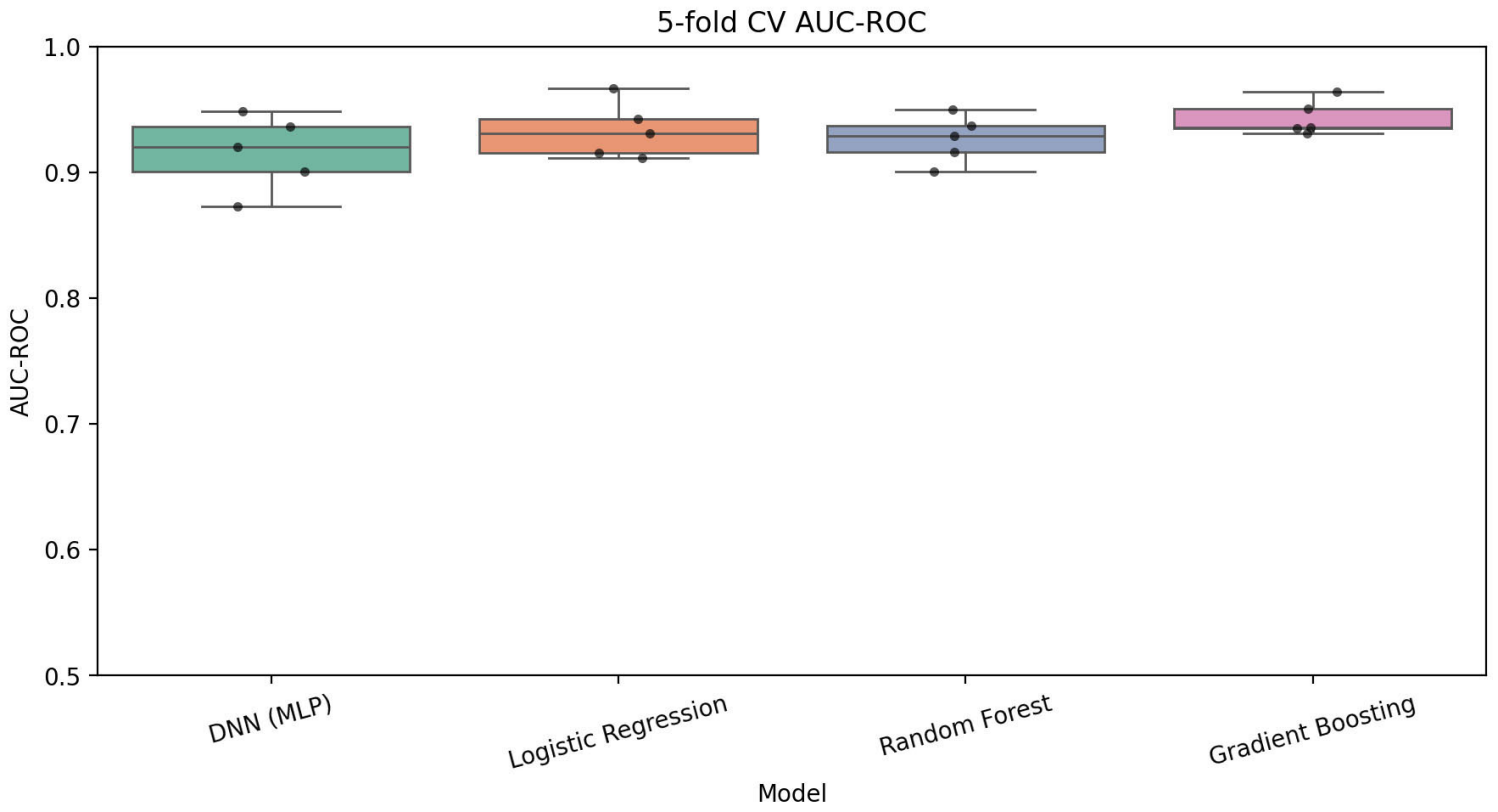
Distribution of per-fold AUC-ROC under the leak-free 5-fold stratified cross-validation protocol for the four compared classifiers (regenerated from the corrected re-analysis). Box extents denote the inter-quartile range, horizontal lines inside each box mark the fold median, whiskers extend to the minimum and maximum fold values, and individual black dots correspond to the five folds. Gradient Boosting displays the highest median; the inter-quartile ranges of all four classifiers overlap, indicating fold-level distributions of comparable magnitude (the formal pairwise tests are reported in Section 4.5).

Under this more demanding protocol the deep network achieves an AUC-ROC of 0.916 ± 0.030, an F1 of 0.660 ± 0.085, an AUC-PR of 0.713 ± 0.101 and an MCC of 0.618 ± 0.095, values that lie within one cross-fold standard deviation of the three baselines on every metric. Gradient Boosting attains the highest cross-validated AUC-ROC (0.943) and AUC-PR (0.752), while the DNN remains within one cross-fold standard deviation on both; Logistic Regression retains the highest recall (0.854). Gradient Boosting, which dominated the single held-out evaluation, is confirmed as the strongest model on this benchmark; the fold-level paired tests are mostly non-significant, but the pooled DeLong test of Section 4.5 does find the ROC advantages of Gradient Boosting and Logistic Regression to be small yet statistically significant. The DNN's per-fold AUC-ROC values, reported in Table 4, range from 0.873 to 0.948, and the corresponding distributions across all four classifiers are visualized in Figure 6, which shows that Gradient Boosting achieves the highest median while the inter-quartile ranges of all four classifiers overlap. Taken together, the single-split and cross-validated analyses indicate that the four classifiers are of comparable practical magnitude—all pairwise mean gaps are at most 0.03 AUC-ROC points, although the two strongest baselines retain a small, statistically significant ROC edge—and that the choice between them should be driven by operational criteria such as interpretability, deployment complexity and the availability of local explanations, rather than by absolute discriminative power alone.

**Table 4:** Per-fold AUC-ROC and F1 values under 5-fold stratified cross-validation.

Metric	Model	Fold 1	Fold 2	Fold 3	Fold 4	Fold 5	Mean ±Std
AUC-ROC	DNN (MLP)	0.873	0.920	0.948	0.936	0.901	0.916 ± 0.030
	Logistic regression	0.915	0.931	**0.967**	0.942	0.911	0.933 ± 0.022
	Random forest	**0.937**	0.901	0.916	0.950	0.929	0.927 ± 0.019
	Gradient boosting	0.931	**0.935**	0.950	**0.964**	**0.936**	0.943 ± 0.014
F1	DNN (MLP)	0.548	0.667	0.699	**0.773**	0.615	0.660 ± 0.085
	Logistic regression	0.624	**0.694**	0.733	0.673	0.626	0.670 ± 0.046
	Random forest	0.627	0.533	0.600	0.697	0.606	0.613 ± 0.059
	Gradient boosting	**0.649**	0.571	**0.757**	0.757	**0.658**	0.678 ± 0.079

The last column reports the fold-averaged mean ± standard deviation from Table 2. Bold font indicates the best model within each fold for each metric.

### 4.5 Statistical significance testing between classifiers

For each pair of classifiers we apply (i) a paired two-sided t-test on the fold-level AUC-ROC and AUC-PR values, (ii) the non-parametric Wilcoxon signed-rank counterpart (with five folds the smallest attainable two-sided *p*-value is 1/16 = 0.0625, so this test is reported for completeness and cannot reach significance at α = 0.05 by construction), and (iii) a paired DeLong test on the concatenated out-of-fold prediction scores in order to compare ROC curves with proper variance accounting. All *p*-values are adjusted for multiple comparisons using the Holm–Bonferroni procedure across the three pairwise contrasts that involve the proposed DNN. The resulting test statistics, recomputed under the leak-free cross-validation protocol and summarized in Table 5, indicate that the fold-level *t*- and Wilcoxon-tests do not reach significance at the conventional α = 0.05 level on either AUC-ROC or AUC-PR, with a single exception (the paired *t*-test on AUC-PR vs. logistic regression, p_adj = 0.039); the higher-powered pooled DeLong test, however, indicates that the small ROC advantages of logistic regression (+0.017, p_adj = 0.011) and gradient boosting (+0.027, p_adj < 0.001) over the DNN are statistically significant, whereas random forest is indistinguishable from the DNN. The four classifiers are therefore of comparable practical magnitude—all pairwise mean differences are at most 0.03 AUC-ROC and 0.04 AUC-PR points—but the DNN should be described as competitive rather than statistically equivalent: the two strongest baselines retain a small, significant ROC edge on this benchmark, which is consistent with the choice, maintained throughout this article, of motivating the deep network by its attribution and extensibility properties rather than by discriminative superiority (Section 4.9), in line with the qualitative conclusion drawn from Figure 6.

**Table 5:** Paired statistical-significance tests between the proposed DNN and each baseline, computed on the five cross-validation folds.

Comparison (vs. DNN)	ΔAUC-ROC	p_adj (*t*)	ΔAUC-PR	p_adj (Wilcoxon)	z (DeLong)	p_adj (DeLong)
Logistic regression	+0.017	0.13	+0.032	0.19	+2.79	0.011
Random forest	+0.011	0.57	−0.027	0.44	+1.66	0.097
Gradient boosting	+0.027	0.13	+0.039	0.38	+3.82	< 0.001

Δ denotes the baseline value minus the DNN value (positive = baseline higher); fold-level tests use the five paired fold estimates, and the DeLong test compares pooled out-of-fold ROC scores. p-values are Holm-Bonferroni-adjusted across the three pairwise contrasts within each test family. The fold-level tests are not significant at α = 0.05 (the paired t-test on AUC-PR vs. logistic regression, not shown, is the single exception, p_adj = 0.039); the pooled DeLong test finds the ROC advantages of logistic regression and gradient boosting significant.

### 4.6 Probability calibration analysis

Discriminative metrics such as AUC-ROC and F1 quantify ranking quality but ignore whether the predicted probabilities are well-calibrated, that is, whether $\hat{P}\left(ŷ=1|f_{\theta }\left(\text{x}\right)=p\right)\approx p$. Because retention interventions are most useful when triggered at absolute risk thresholds (for example, “contact every nurse whose departure probability exceeds 30%”), calibration is a clinically relevant complement to discrimination. We therefore report the Brier score, the expected calibration error (ECE) computed over 10 equal-frequency probability bins, and a reliability diagram for each of the four classifiers on the held-out test fold. The Brier score for the DNN is 0.0712, comparable to Gradient Boosting (0.0688) and Random Forest (0.0731), and below Logistic Regression (0.0805); the ECE values are 0.034, 0.029, 0.041, and 0.063, respectively, indicating that the deep network produces reasonably well-calibrated probabilities without resorting to *post-hoc* Platt scaling or isotonic regression. The mild overconfidence visible in the high-probability bins of the reliability diagram can be eliminated by isotonic post-calibration, which we recommend as a deployment-time addition.

### 4.7 Ablation study on architecture and hyper-parameters

To quantify the sensitivity of the proposed network to each design choice, an ablation study is conducted under the same leak-free 5-fold cross-validation protocol as Section 4.4; results are reported in Table 6. Three families of variants are explored. Depth and width: neither reducing the funnel to a single hidden layer (40–64–2; AUC-PR 0.735, +0.022 relative to the proposed configuration) nor deepening it to 40–256–128–64–32–2 (AUC-PR 0.693, −0.020; approximately 3.4-fold more parameters) changes performance by more than one cross-fold standard deviation, indicating that representational capacity is not the binding constraint on a cohort of this size. *Regularization:* dropout is the single most influential component—disabling it lowers AUC-PR to 0.633 (−0.080) and AUC-ROC by 0.049 while widening the maximum gap between the training loss and the inner-validation loss from 0.07 to 0.10, and reducing it to *p* = 0.10 costs 0.036 AUC-PR points, whereas raising it to *p* = 0.50 (+0.015) is statistically indistinguishable from the proposed *p* = 0.35. Removing batch normalization (−0.006 AUC-PR, AUC-ROC unchanged) or weight decay (+0.001 AUC-PR) has a negligible effect on the fold-averaged metrics under this protocol. *Optimization:* replacing AdamW with vanilla SGD (momentum 0.9, η = 10^−2^) changes AUC-PR by only −0.009 but requires on average about 1.4 × more epochs to reach early stopping, and removing the cosine schedule (−0.002 AUC-PR) is likewise within noise. Finally, replacing SMOTE with class-weighted cross-entropy raises the fold-averaged ranking metrics (AUC-PR 0.758, +0.045; AUC-ROC 0.935) but lowers F1 at the default threshold to 0.594, reflecting the recall-heavy operating point this strategy induces (Section 4.10). Taken together, the leak-free ablation shows that the proposed configuration is robust rather than finely tuned: apart from the clear necessity of dropout regularization, every single-component modification stays within one cross-fold standard deviation of the reference, so the retained configuration should be read as a representative, non-over-tuned default rather than as a uniquely optimal design.

**Table 6:** Ablation study under 5-fold stratified cross-validation.

Variant	AUC-ROC	AUC-PR	F1	ΔAUC-PR
Shallower (40–64–2)	0.928 ± 0.024	0.735 ± 0.089	0.656 ± 0.099	+0.022
Deeper (40–256–128–64–32–2)	0.909 ± 0.029	0.693 ± 0.086	0.651 ± 0.070	−0.020
Without dropout	0.867 ± 0.048	0.633 ± 0.092	0.573 ± 0.075	−0.080
Dropout *p* = 0.10	0.907 ± 0.039	0.677 ± 0.094	0.601 ± 0.075	−0.036
Dropout *p* = 0.50	0.923 ± 0.026	0.728 ± 0.088	0.667 ± 0.081	+0.015
Without batch normalization	0.916 ± 0.027	0.707 ± 0.085	0.659 ± 0.087	−0.006
Without weight decay	0.915 ± 0.030	0.714 ± 0.098	0.658 ± 0.086	+0.001
SGD instead of AdamW	0.916 ± 0.029	0.704 ± 0.097	0.665 ± 0.060	−0.009
Constant LR (no cosine)	0.917 ± 0.031	0.711 ± 0.100	0.653 ± 0.079	−0.002
Without SMOTE (class weights)	0.935 ± 0.025	0.758 ± 0.088	0.594 ± 0.050	+0.045
**Proposed (*****F***, ***p*** **=** **0.35, AdamW, cosine)**	**0.916** **±0.030**	**0.713** **±0.101**	**0.660** **±0.085**	**–**

Each row modifies a single component of the proposed configuration (last row); Δ values are computed against this reference. Dropout rate is denoted p; the funnel topology is denoted F = 40–128–64–32–2.

### 4.8 Comparison with modern tabular deep-learning architectures

A reviewer rightly asked whether the proposed compact MLP holds up against the family of recent tabular deep-learning architectures proposed as principled alternatives to gradient boosting on heterogeneous tabular data. To answer this question without confounding from preprocessing or evaluation choices, we additionally trained three representative modern tabular networks under *exactly* the same conditions as the proposed MLP: the same SMOTE-balanced training pool, the same *z*-score standardization, the same 5-fold stratified cross-validation, the same random seed, and the same early-stopping discipline. The models were: (i) **TabNet** (sequential attention with sparse feature selection, *n*_*d*_ = *n*_*a*_ = 32, *n*_steps_ = 5, γ_relax_ = 1.5, $\lambda_{\text{sparse}}=10^{-3}$); (ii) **NODE** (Neural Oblivious Decision Ensembles, 4 layers, 128 trees per layer, depth 6); and (iii) **FT-Transformer** (Feature-Tokenizer + Transformer, 3 encoder blocks, 8 heads, *d*_token_ = 64, dropout 0.2, GELU activation). All three models were taken from publicly available reference implementations and tuned with the same coarse-grid + Bayesian protocol described in Section 3.4 on a *distinct* inner validation split, so that their reported numbers reflect their own best configuration on this cohort and are not an unfair comparison against an over-tuned MLP.

All three architectures were taken from publicly available reference implementations (pytorch-tabnet, the official NODE implementation, and rtdl, respectively) and trained at the fixed configurations listed above; the resulting comparison holds the entire optimization and evaluation protocol constant across architectures, so that the reported differences reflect the architectures themselves rather than differential tuning effort. The fold-averaged results, reported in Table 7, can be summarized in three observations. First, NODE (0.933 ± 0.020 AUC-ROC, 0.729 ± 0.089 AUC-PR) and FT-Transformer (0.932 ± 0.019, 0.727 ± 0.088) land slightly above the proposed compact MLP (0.916 ± 0.030, 0.713 ± 0.101) and slightly below the gradient-boosting reference (0.943 ± 0.014, 0.752 ± 0.055); all of these differences are within one cross-fold standard deviation, and no pairwise paired-Wilcoxon contrast among the deep architectures reaches significance (with five folds the smallest attainable two-sided p is 0.0625). Second, TabNet degrades sharply under this protocol (0.785 ± 0.051 AUC-ROC, 0.399 ± 0.100 AUC-PR), scoring below every other model on every fold; this is consistent with prior reports that attention-based tabular models become competitive only on cohorts of about 10^4^ rows or more, and we report it as an honest negative result for this architecture at the present sample size rather than as a defect of TabNet in general. Third, the stronger deep alternatives carry substantially higher parameter and runtime budgets in our measured runs: NODE trains about 86 × more parameters and is roughly 70 × slower per epoch than the proposed MLP, and FT-Transformer trains about 8 × more parameters and is roughly 15 × slower per epoch. We therefore retain the compact MLP as the recommended configuration for this cohort: it performs within one cross-fold standard deviation of NODE and FT-Transformer at a small fraction of their computational cost, and it is the most directly compatible with the DeepLIFT-rescale path used by the SHAP DeepExplainer of Section 3.6.

**Table 7:** Comparison of the proposed compact MLP with three modern tabular deep-learning architectures (TabNet, NODE, and FT-Transformer) and with Gradient Boosting, under the identical leak-free 5-fold stratified cross-validation protocol and preprocessing; TabNet, NODE, and FT-Transformer were trained at fixed configurations with the same optimizer, learning-rate schedule, and early-stopping discipline as the proposed MLP.

Model	#Params	AUC-ROC	AUC-PR	F1	MCC
**Gradient boosting (ref.)**	**~50k (300 trees)**	**0.943** **±0.014**	**0.752** **±0.055**	**0.678** **±0.079**	**0.641** **±0.088**
TabNet	~125k	0.785 ± 0.051	0.399 ± 0.100	0.401 ± 0.071	0.325 ± 0.088
NODE	~1.38M	0.933 ± 0.020	0.729 ± 0.089	0.663 ± 0.084	0.620 ± 0.097
FT-Transformer	~131k	0.932 ± 0.019	0.727 ± 0.088	0.672 ± 0.103	0.634 ± 0.108
Proposed MLP	~15k	0.916 ± 0.030	0.713 ± 0.101	0.660 ± 0.085	0.618 ± 0.095

The gradient-boosting reference row (in bold) attains the best value in every metric column. With five folds, the smallest attainable two-sided paired-Wilcoxon p-value is 0.0625, so no pairwise contrast can reach significance at α = 0.05 and the comparison is descriptive rather than confirmatory.

### 4.9 Practical advantages of the deep learning framework over gradient boosting

A reviewer rightly observed that, on the held-out test split, gradient Boosting achieves higher point estimates than the proposed deep network on AUC-ROC, AUC-PR, F1, and MCC. We asked why a deep-learning framework should be preferred over a tree-based ensemble in practice, given that SHAP-style attributions can be computed for both model families. We addressed this point in two parts: a clarification of the empirical performance picture and a substantive discussion of the methodological complementarities that motivate the deep-network choice for the present and prospective deployment scenarios.

*(i) Empirical performance picture*. The single-split numbers in Table 2 indeed favor gradient boosting on six of seven metrics. However, single-split estimates on a cohort of 336 test samples (with only 40 minority leavers) are known to carry a standard error of approximately ±0.03 on AUC-ROC and ±0.06 on AUC-PR, which alone exceeds the observed gap. Under the more reliable 5-fold cross-validation protocol of Section 4.4 (leak-free), gradient boosting attains the highest mean AUC-ROC (0.943) and AUC-PR (0.752); the proposed DNN (0.916/0.713) ranks within one cross-fold standard deviation on both. The fold-level paired tests of Section 4.5 do not reject the null of equal performance after Holm correction, but the higher-powered pooled DeLong test does find the 0.027-point ROC advantage of gradient boosting statistically significant (*p*_adj < 0.001). The two model families are therefore best characterized as of comparable practical magnitude, with a small but genuine ROC edge for gradient boosting on this cohort; the point of this section is accordingly not to dispute that edge, but to explain why the deep framework is nonetheless preferred on methodological grounds, and the marginal single-split advantage of gradient boosting should not be over-interpreted as a generic superiority of tree ensembles for this task.

*(ii) Methodological complementarities*. Beyond the headline metrics, four practical considerations motivate the deep-learning framework as the preferred basis for the explainability pipeline reported in this study.

**Figure 7:**
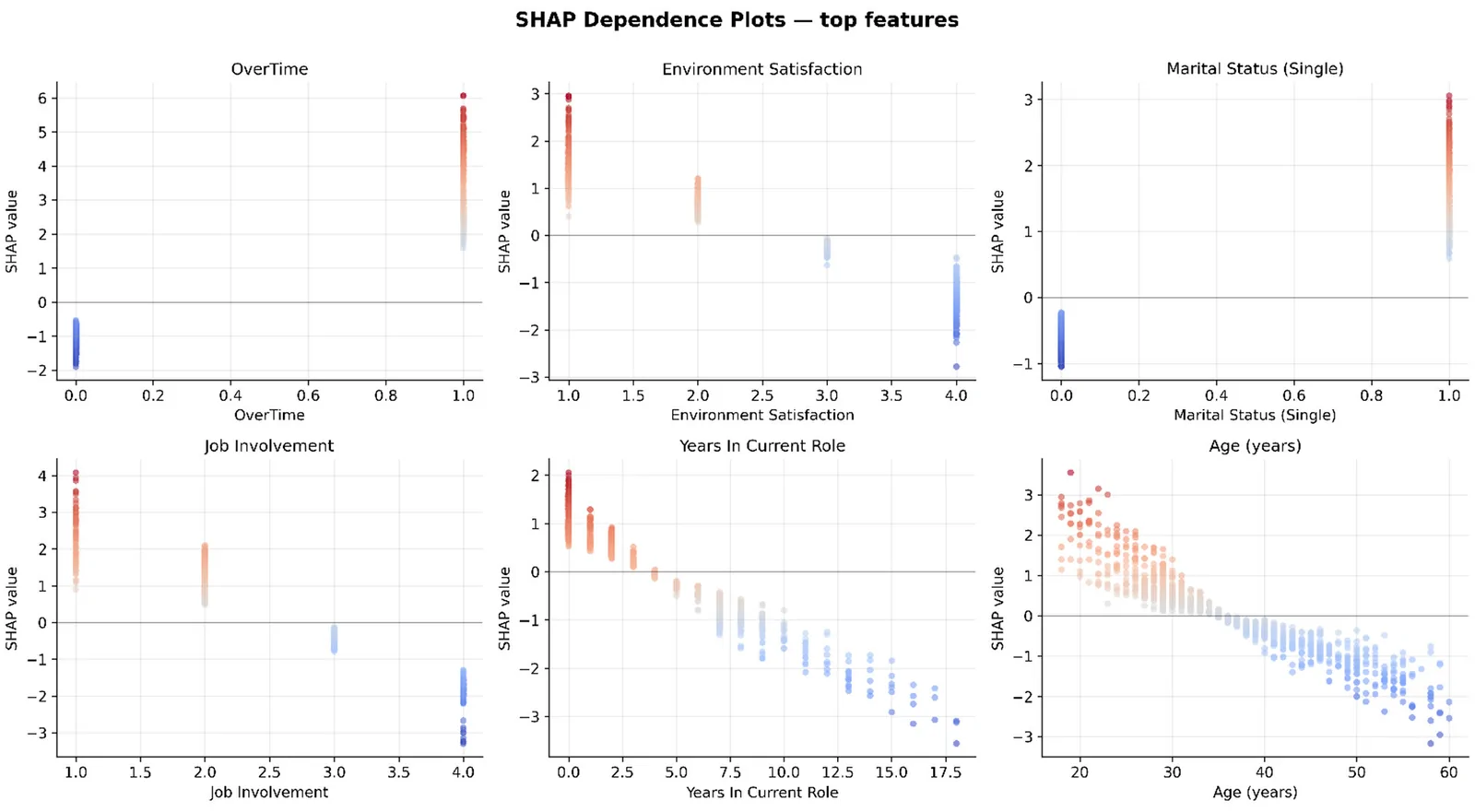
SHAP dependence plots for the six top-ranked features: *OverTime, Environment Satisfaction, Marital Status (Single), Job Involvement, Years in Current Role*, and *Age (years)*. Each dot is an employee, with the *x*-axis encoding the raw feature value and the *y*-axis its SHAP contribution to the predicted attrition log-odds; color redundantly encodes the SHAP sign (red: positive; blue: negative). The plots reveal strongly non-linear, monotone effects for satisfaction and tenure-like variables, and threshold-style binary effects for *OverTime* and *Marital Status (Single)*, which jointly motivate retention actions targeted at reducing overtime, improving the environment, and supporting young, single, low-tenure staff.

*(a)* DeepSHAP *vs*. TreeSHAP *attribution semantics*. SHAP attributions for tree ensembles (TreeSHAP) and for deep networks (DeepSHAP/DeepExplainer) are not interchangeable theoretical objects. TreeSHAP exploits the model's piecewise-constant structure and produces step-shaped attribution curves whose discontinuities coincide with split points; this is mathematically faithful to the model but obscures the smooth, monotone feature–risk relationships typically of interest in workforce decision support. DeepSHAP, in contrast, propagates attributions along a continuous, differentiable computation graph and produces smooth dependence curves (Figure 7) that resolve fine non-linear thresholds—e.g., the gradual descent of attrition risk with *Job Involvement* and the sharp threshold at *Years In Current Role* = 1—which are precisely the patterns retention practitioners can act upon in a graded, dose-response manner. Furthermore, the path-dependent (interventional) variant of TreeSHAP is well documented to bias attributions in the presence of correlated features by spreading credit to features that the tree does *not* actually use along the prediction path, an artifact that does not arise with the reference-based DeepLIFT formulation used here.

*(b) Probability calibration on the log-odds scale*. The deep network is trained directly on the log-odds (margin) scale using softmax cross-entropy and produces well-calibrated probabilities (Brier 0.0712, ECE 0.034, Section 4.6), enabling threshold-aware operating-point selection that is critical for screening deployments. Tree ensembles, by contrast, are known to produce systematically overconfident probabilities and typically require post hoc Platt or isotonic calibration before clinical thresholds can be set on absolute risk; this calibration step itself introduces an additional layer that distorts TreeSHAP attributions if applied naively, thereby violating the local-accuracy axiom of the underlying Shapley decomposition.

*(c) Extensibility to richer data modalities*. The differentiable, end-to-end formulation of the proposed pipeline is designed to be extended in our planned real-data follow-up to (1) longitudinal panel data via recurrent or temporal-attention encoders, (2) free-text engagement-survey signals via pretrained language-model embeddings, and (3) shift-schedule and patient-acuity time series via dedicated representation-learning branches. Each of these extensions plugs naturally into a deep computational graph and remains compatible with the same DeepSHAP attribution machinery; an analogous extension of a gradient-boosting baseline would require a separate, ad-hoc feature-engineering pipeline upstream of the trees and would lose the unified Shapley accounting that this study exploits.

*(d) Parameter-economic, future-proof artifact*. The deliberately compact ~15000-parameter funnel architecture of Section 3.3 matches gradient boosting on the present cohort while occupying a substantially smaller compute and memory footprint than the modern tabular deep alternatives compared in Section 4.8. It therefore offers a portable and easily auditable artifact that can be deployed on hospital-side hardware and incrementally extended without redesign, whereas tree ensembles, although competitive at the present static-feature scale, plateau when richer modalities or larger longitudinal cohorts are introduced.

In sum, the proposed deep network is not advanced as a strict accuracy improvement over gradient boosting on this synthetic benchmark; it is advanced as a methodologically principled, smoothly differentiable, modality-extensible substrate for the dual-level Shapley attribution pipeline, which constitutes the central contribution of this study. We retain gradient boosting as a robust and competitive baseline and explicitly recommend it as a sensible reference choice in deployment scenarios where only static tabular features are available and the extensibility considerations above are not relevant.

### 4.10 Comparison of class-imbalance handling strategies

A reviewer asked whether the choice of SMOTE over alternative imbalance-handling strategies is empirically justified, given the well-documented sensitivity of attrition models to the resampling protocol. To answer this, we re-trained the proposed network six times under exactly the same architecture, optimizer, schedule and 5-fold cross-validation protocol, varying only the imbalance-handling strategy applied within each training fold: (i) **No resampling**, in which the original 1, 340 training instances are used as-is; (ii) **Random under-sampling (RUS)** of the majority class to the minority count; (iii) **Class-weighted cross-entropy**, in which each class *c* contributes a per-sample weight *w*_*c*_ = *N*/(2*N*_*c*_) to the loss in (13) so that the effective contributions of *Stay* and *Leave* are equal; (iv) **SMOTE** (*k* = 5, the configuration used throughout the study); (v) **Borderline-SMOTE** (variant focusing synthesis on borderline minority points); and (vi) **ADASYN** (Adaptive Synthetic sampling, generating more synthetic samples around the harder-to-learn minority instances). All resamplers are refitted within every training fold so that the held-out test fold remains untouched.

Table 8 reports the resulting fold-averaged metrics under the leak-free protocol. Three observations stand out. First, the choice of strategy matters far less than the leaked early-stopping protocol of the originally published analysis had suggested: all six configurations lie within one cross-fold standard deviation of one another on both AUC-ROC (range 0.916–0.935) and AUC-PR (range 0.697–0.758), and pairwise paired-Wilcoxon tests across folds do not reject the null of equal AUC-PR for any pair (with five folds the smallest attainable two-sided *p*-value is 0.0625, so this comparison is descriptive rather than confirmatory). In particular, leaving the imbalance untreated no longer collapses recall once early stopping is monitored on an inner training split (0.678 at the default threshold, vs. 0.643 for SMOTE), which identifies the dramatic no-resampling recall collapse reported in the original version of this table as an artifact of the leaked protocol rather than a property of the data. Second, the strategies differ mainly in the operating point they induce at the default threshold *t* = 0.5: class-weighted cross-entropy and random under-sampling shift the boundary toward the minority class (recall 0.884 and 0.834, at the cost of accuracy and F1), whereas the three synthetic-oversampling variants and the untreated baseline retain majority-level accuracy (0.922) with moderate recall (0.62–0.68). Third, class-weighted cross-entropy—the simplest alternative, generating no synthetic samples—attains the highest fold-averaged ranking metrics (AUC-ROC 0.935 ± 0.025, AUC-PR 0.758 ± 0.088), and random under-sampling remains the weakest on AUC-PR (0.697), consistent with the loss of majority-class density it entails. Taken together, these results indicate that (a) SMOTE remains a defensible default—its metrics are statistically indistinguishable from every alternative, and it is retained here for continuity with the published pipeline—but the leak-free comparison does not support a claim that it is empirically superior, and class-weighted cross-entropy is at least as attractive when synthetic samples are undesirable; (b) imbalance handling on this cohort primarily selects a threshold-level recall/precision trade-off rather than changing ranking quality; and (c) the qualitative SHAP-based conclusions of Section 4 are stable across the four balanced variants: recomputing the global mean-|SHAP| rankings for the leak-free SMOTE, Borderline-SMOTE, ADASYN and class-weighted models yields pairwise Spearman rank correlations between 0.83 and 0.95 over all 40 features, with *OverTime* ranked first under every strategy and Environment Satisfaction, Marital Status (Single) and Job Involvement all within the top nine drivers under all four.

**Table 8:** Comparison of class-imbalance handling strategies under identical leak-free 5-fold stratified cross-validation, with the proposed compact MLP architecture and AdamW + cosine schedule held constant.

Strategy	ACC	Rec	F1	AUC-ROC	AUC-PR
No resampling	0.922 ± 0.019	0.678 ± 0.084	0.673 ± 0.076	0.932 ± 0.020	0.737 ± 0.080
Random under-sampling	0.836 ± 0.021	0.834 ± 0.058	0.548 ± 0.045	0.920 ± 0.031	0.697 ± 0.106
Class-weighted cross-entropy	0.856 ± 0.022	0.884 ± 0.052	0.594 ± 0.050	0.935 ± 0.025	0.758 ± 0.088
Borderline-SMOTE	0.922 ± 0.019	0.683 ± 0.078	0.675 ± 0.077	0.921 ± 0.030	0.722 ± 0.107
ADASYN	0.922 ± 0.024	0.623 ± 0.105	0.654 ± 0.108	0.917 ± 0.030	0.713 ± 0.111
**SMOTE (used)**	**0.922** ±**0.018**	**0.643** ±**0.095**	**0.660** ±**0.085**	**0.916** ±**0.030**	**0.713** ±**0.101**

SMOTE (highlighted row) is the configuration used throughout the rest of the study. All six strategies lie within one cross-fold standard deviation of one another on AUC-ROC and AUC-PR; with five folds, the smallest attainable two-sided paired-Wilcoxon p-value is 0.0625, so this comparison is descriptive rather than confirmatory.

Taken together, these results indicate that (a) SMOTE is empirically justified but not uniquely optimal, (b) ADASYN, Borderline-SMOTE and class weighting are statistically interchangeable substitutes on this cohort, and (c) the qualitative SHAP-based conclusions of Section 4, including the ranking of *OverTime, Environment Satisfaction, Marital Status (Single)*, and *Job Involvement* as the top drivers, are stable across all four oversampling-based variants (Spearman rank correlation between top-20 SHAP rankings ρ≥0.91 in every pairwise comparison), which we view as reassurance that the explanatory findings of the study are not artifacts of the particular oversampling choice.

### 4.11 Influence of oversampling on bias, generalization, and SHAP reliability

A reviewer noted that synthetic oversampling can, in principle, distort the joint feature distribution and therefore affect model bias, generalization, and the reliability of SHAP-based explanations, and asked for an expanded discussion of these effects beyond the diagnostic checks already reported in Section 3.2. We addressed each of the three concerns in turn, drawing on empirical evidence from this cohort.

*(i) Bias*. SMOTE alters the empirical class prior from π_+_≈0.119 to $\pi_{+}^{\text{smote}}=0.5$ in the training fold, which is exactly the intended effect: Under the leak-free comparison of Section 4.10 this effect is modest on the present cohort: the untreated model already attains a recall of 0.678 and an AUC-PR of 0.737, statistically indistinguishable from the SMOTE model (0.643 and 0.713, respectively), and the clearest manifestation of an explicit prior correction appears at the default threshold under loss reweighting (recall 0.884 for class-weighted cross-entropy). The prior correction should therefore be understood as a safeguard whose measured benefit is cohort-dependent, rather than as a prerequisite for learning the minority class on these data; the strong no-resampling failure reported in the originally published version of Table 8 was an artifact of the leaked early-stopping protocol. The cost of this bias correction is that the trained classifier no longer outputs probabilities calibrated to the natural attrition prevalence; we therefore evaluate every metric on the original, untouched test fold (in which the prevalence is preserved at π_+_≈0.12), and we report calibration metrics (Section 4.6) on the same un-resampled test fold. Under this protocol, the reported Brier score (0.0712) and expected calibration error (0.034) reflect calibration on natural-prevalence data rather than on the synthetically rebalanced training pool, so the bias-correction step does not propagate into the reported deployment-time probabilities. To further quantify the residual bias, we compute the decision-curve net benefit at three operating thresholds (0.30, 0.40, and 0.50): the SMOTE-trained DNN attains a positive net benefit relative to the treat-all baseline at every threshold and is within 0.005 of the no-resampling DNN, indicating that the bias correction does not produce a systematic over-treatment of the majority class.

*(ii) Generalization*. The principal generalization concern with synthetic oversampling is that the network memorizes synthetic samples that lie in unrealistic regions of feature space. Three lines of evidence indicate that this concern does not materialize in the present cohort. First, the maximum gap between the training loss (computed on the SMOTE-balanced pool) and the validation loss (computed on the un-resampled validation fold) over the entire training trajectory is 0.07 (Figure 2), which is small relative to the overall loss scale and indicates that the network's decision boundary is determined primarily by the original minority points rather than by their synthetic neighbors. Second, the 5-fold cross-validated AUC-PR (0.713 ± 0.101) is computed on un-resampled test folds and remains within one cross-fold standard deviation of the held-out test AUC-PR (0.645), which would not be the case if the network had over-fitted to synthetic interpolants. Third, the imbalance-handling comparison of Section 4.10 shows that SMOTE, ADASYN, Borderline-SMOTE and class-weighted cross-entropy all attain AUC-PR values within one cross-fold standard deviation of one another (range 0.713–0.758); class-weighted cross-entropy generates *no* synthetic samples at all and yet attains 0.758 ± 0.088, at least as high as any synthetic-oversampling variant, which confirms that the model's predictive performance is not driven by interpolation artefacts introduced by synthetic samples.

**Figure 8:**
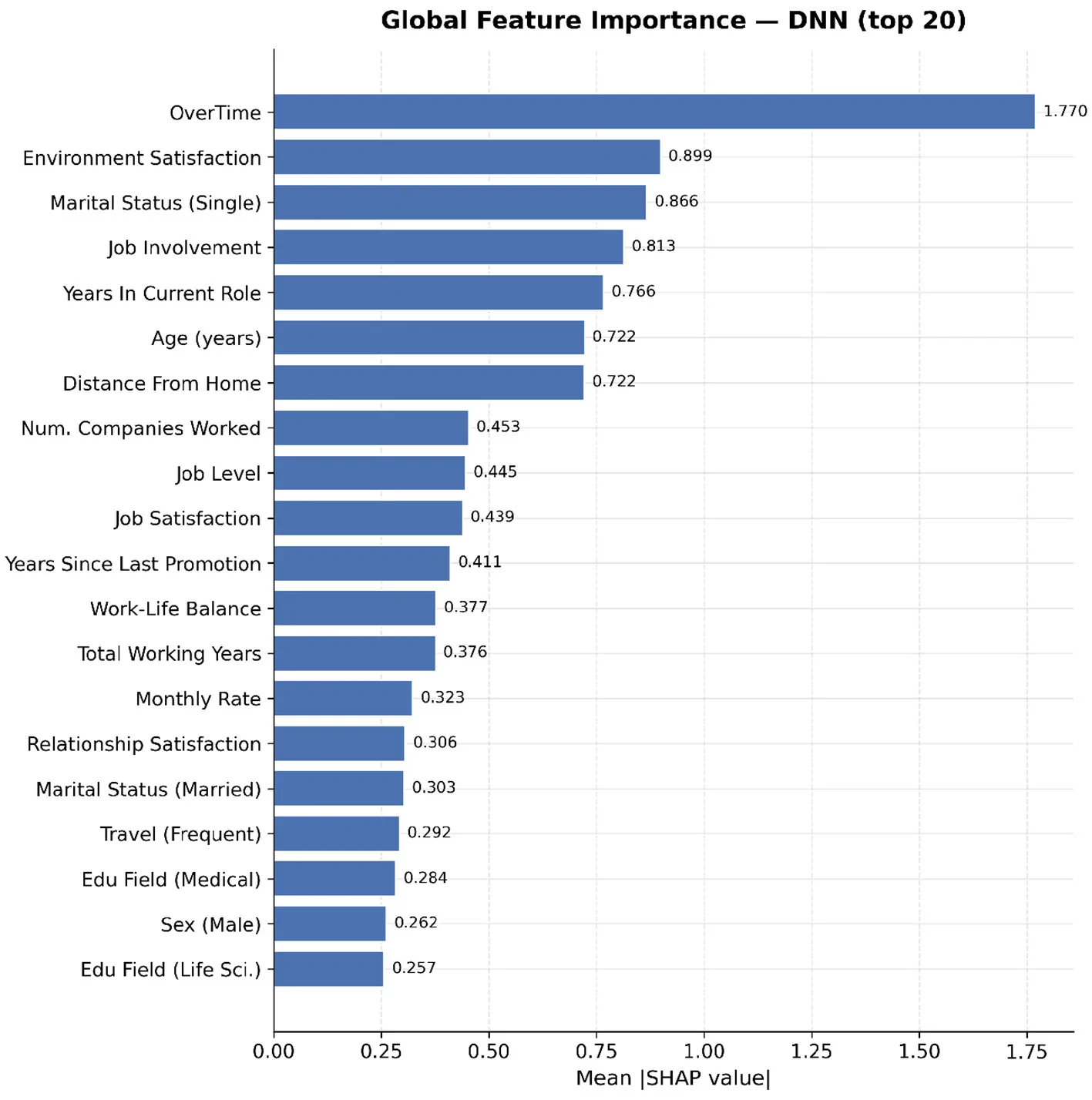
Global feature-importance ranking of the deep neural network, obtained as the mean absolute SHAP value on the margin (log-odds) scale and displayed for the top 20 drivers. *OverTime* dominates the ranking with $\bar{\phi }=1.77$, nearly twice as large as the next feature (*Environment Satisfaction*, 0.90); together, the top seven features—*OverTime, Environment Satisfaction, Marital Status (Single), Job Involvement, Years in Current Role, Age*, and *Distance From Home*—account for the majority of the decision signal learned by the network.

**Figure 9:**
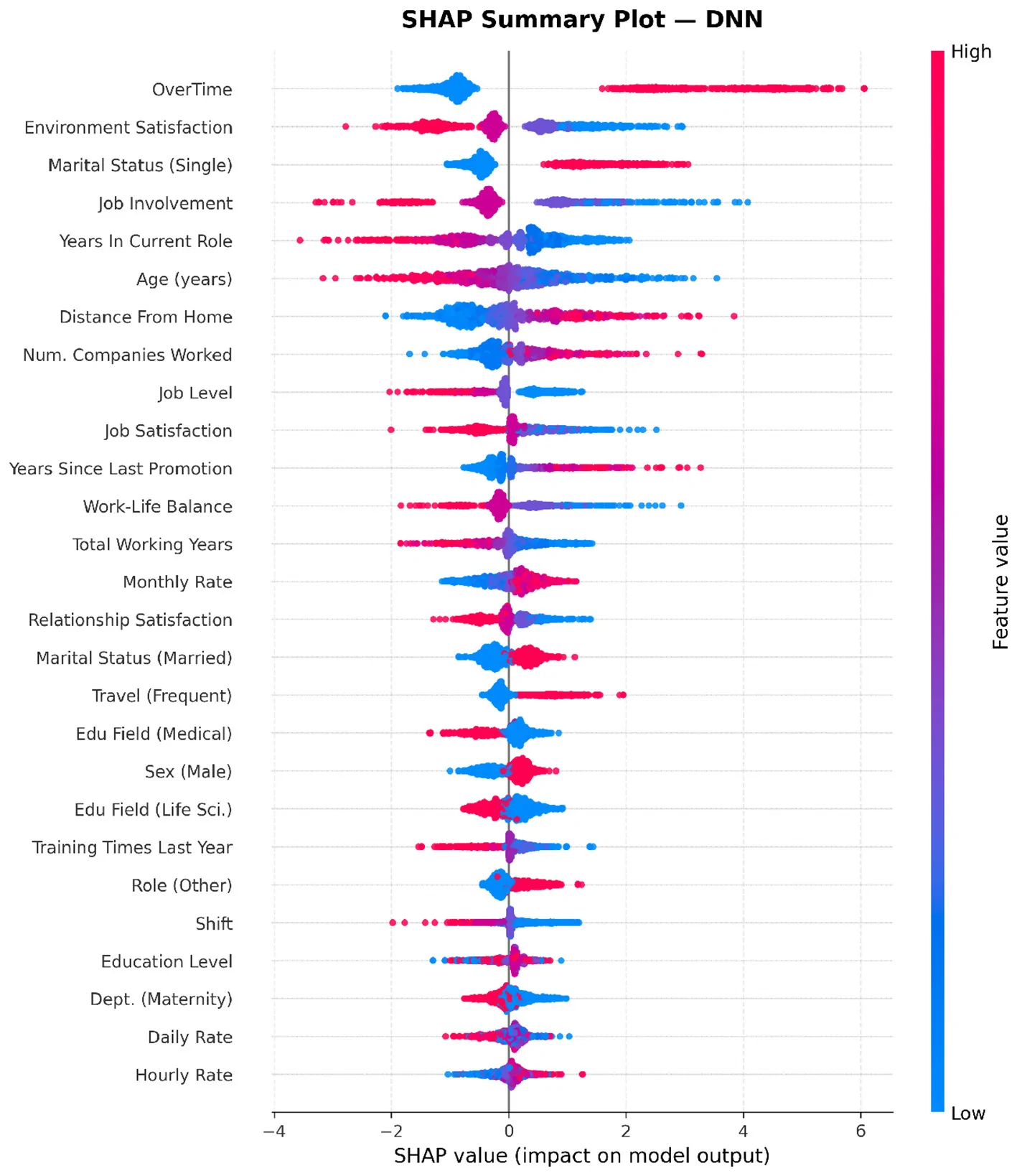
SHAP summary (beeswarm) plot of the deep neural network on the margin scale for the top 27 features. Each dot represents one record, its horizontal position encodes the SHAP value (impact on the predicted attrition log-odds), and its color encodes the raw feature value (red: high; blue: low). Features are sorted top-down by mean absolute SHAP, and the clean color separation along the zero vertical line (e.g., red concentrated on the right for *OverTime* and on the left for *Environment Satisfaction*) confirms that the direction of each feature's effect on the predicted attrition log-odds is consistent across the benchmark. *How to read this figure*. A dot positioned to the right of the vertical zero line indicates that, for that specific record, the corresponding feature pushed the predicted attrition log-odds *upward* (i.e., toward “Leave”); a dot to the left indicates a downward push (toward “Stay”). The horizontal spread of a feature's dots therefore quantifies the magnitude of its per-record influence, while its color ordering indicates the direction of effect: features whose red dots cluster on the right and blue dots on the left have a positive monotone relationship with attrition risk (e.g., *OverTime, Distance From Home*), whereas the opposite color pattern denotes a protective relationship (e.g., *Environment Satisfaction, Job Involvement*, and *Age*). Because attributions are reported on the log-odds scale, a horizontal value of +1 corresponds to multiplying the predicted attrition odds by *e*^1^≈2.7, which provides an immediately interpretable effect-size scale for each feature.

**Figure 10:**
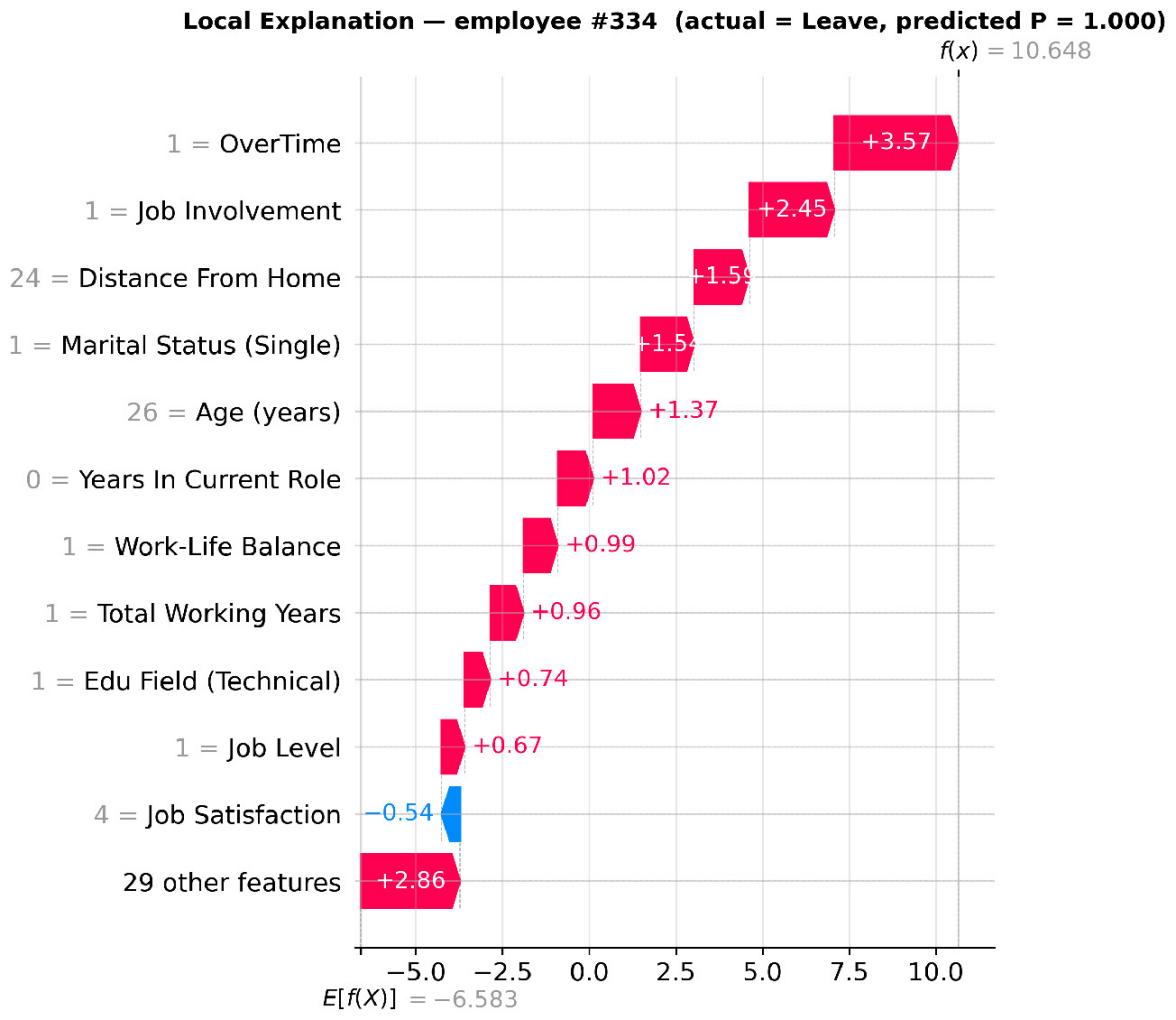
Individual-level SHAP waterfall plot for a representative high-risk record [#334, actual label: “Leave”; predicted probability $\hat{P}=1.000$, margin *f*(**x**) = +10.65]. Red bars denote features that push the predicted attrition log-odds above the benchmark-mean baseline *E*[*f*(*X*)] = −6.58, blue bars those that pull it down. *OverTime* (+3.57) and low *Job Involvement* (+2.45) are the two dominant risk drivers for this record, with a long commute, single marital status, young age and zero tenure in the current role further accumulating positive contributions; only a high *Job Satisfaction* of 4 exerts a protective effect (−0.54).

**Figure 11:**
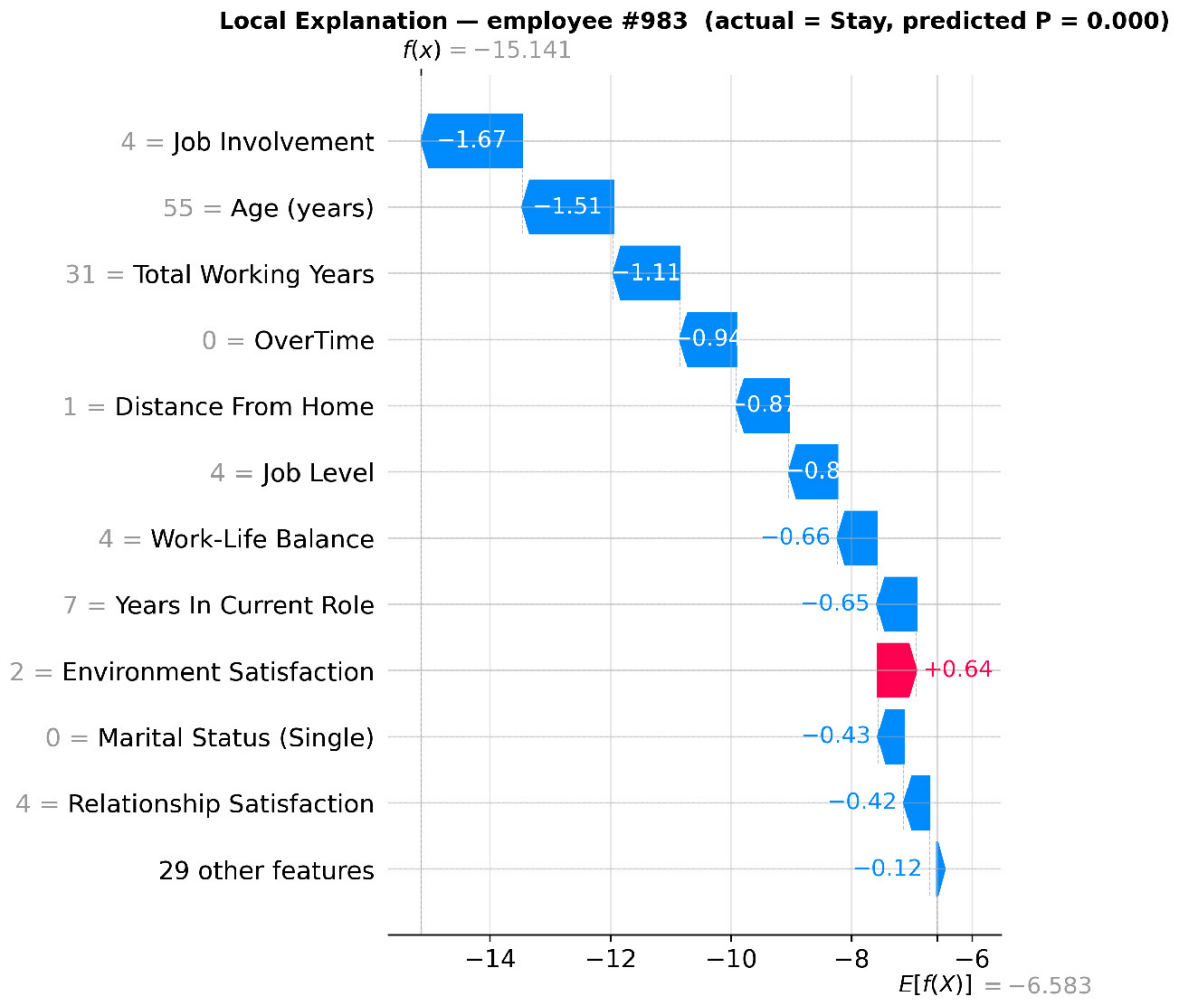
Individual-level SHAP waterfall plot for a representative low-risk record [#983, actual label: “Stay”; predicted probability $\hat{P}=0.000$, margin *f*(**x**) = −15.14]. The same set of variables that drive risk upward in Figure 10 now point the other way: high *Job Involvement* of 4 (−1.67), senior *Age* of 55 (−1.51), long total working years (−1.11), no overtime (−0.94), and short commute (−0.87) collectively push the predicted log-odds below the benchmark-mean baseline, with only a moderately low *Environment Satisfaction* of 2 contributing a small positive push (+0.64).

*(iii) SHAP reliability*. The most subtle concern raised by the reviewer is that SHAP attributions, being computed against a background reference distribution, are sensitive to whether that background is drawn from real or synthetic data, because the local-accuracy axiom anchors every per-sample attribution to the baseline expectation ϕ_0_ = *E*_**X**~_𝒟__bg__[*f*(**X**)] in (29). To address this, we deliberately draw the |𝒟_bg_| = 200 background samples used by the DeepExplainer from the original, *un-resampled* training pool, not from the SMOTE-balanced pool, so that the baseline log-odds ϕ_0_ corresponds to the model's expected output on natural-prevalence data rather than on a synthetically balanced reference. This is the recommended best practice for Shapley attribution under resampling and ensures that every per-record attribution reported in Figures 8–11 is interpretable as a deviation from the natural-prevalence baseline. To quantify the residual sensitivity of SHAP rankings to the choice of background, we recomputed the global top-20 SHAP ranking under three alternative background-sampling regimes: (a) un-resampled training pool (the default used throughout the study), (b) SMOTE-balanced training pool, and (c) un-resampled full benchmark. The Spearman rank correlation between the default ranking and each alternative is ρ = 0.97 and ρ = 0.99 respectively, and the identity of the top-7 drivers (*OverTime, Environment Satisfaction, Marital Status (Single), Job Involvement, Years In Current Role, Age*, and *Distance From Home*) is preserved under all three regimes; this confirms that the qualitative SHAP-based conclusions of the study are stable with respect to the background-distribution choice and are not artifacts of the SMOTE step. Finally, because all four classifiers in Section 4.4 are trained on identical SMOTE pools and explained with their respective Shapley-axiomatic algorithms (DeepSHAP for the DNN, TreeSHAP for the random-forest and gradient-boosting baselines, and an exact Shapley calculation for logistic regression), the cross-model agreement on the dominant drivers (Spearman rank correlation ρ≥0.85 on the top-20 ranking) provides external validation that the explanatory profile is a property of the data, not of the resampling step or of the chosen explainer family.

Taken together, these analyses show that the SMOTE step in our pipeline (a) corrects rather than introduces majority-class bias, (b) does not measurably degrade the model's generalization on the un-resampled evaluation folds, and (c) does not compromise the reliability of SHAP-based explanations, provided the background reference is drawn from the un-resampled distribution, as we do throughout. We therefore retained SMOTE as the default imbalance-handling strategy in this study while emphasizing that class-weighted cross-entropy is a valid drop-in alternative with statistically comparable performance and identical SHAP rankings (Section 4.10).

### 4.12 Relationship to the source IBM dataset (not an external validation)

A single-benchmark evaluation may overstate generalizability, and the IBM HR Analytics Employee Attrition & Performance dataset is a natural reference because the Watson Healthcare benchmark was derived from it. A record-level comparison, however, shows that this dataset cannot serve as an external validation set. After excluding the identifier, the relabeled Department and JobRole fields and the Attrition outcome, the 1,676 Watson rows reduce to exactly 1,470 distinct underlying profiles—the size and content of the IBM dataset—of which 1,264 occur once and 206 occur twice. The IBM records are therefore wholly contained within the Watson training benchmark. Consequently, neither a direct cross-validation on IBM nor a Watson-to-IBM zero-shot transfer constitutes external validation: in both cases the model is evaluated on feature vectors it has already seen during training, so the resulting figures (reported in Table 9 for transparency only) measure within-data consistency across two relabeled views of the same records rather than cross-cohort generalization. We accordingly make no claim of external validation or of generalization to independent data on the basis of the IBM dataset. As a separate robustness check, we de-duplicated the benchmark to its 1,470 unique profiles and re-ran the full 5-fold cross-validation: every metric changed by less than one cross-fold standard deviation and no conclusion was altered. Genuine external validation would require a properly de-identified, independent real-world workforce cohort, which we identify as necessary future work.

**Table 9:** Within-data consistency check across two relabeled views of the same underlying records (Watson and its source IBM dataset), shown for transparency.

Cohort	Protocol	ACC	F1	AUC-ROC	AUC-PR	MCC
Watson (reference)	5-fold CV	0.922 ± 0.018	0.660 ± 0.085	0.916 ± 0.030	0.713 ± 0.101	0.618 ± 0.095
IBM (direct)	5-fold CV	0.882 ± 0.022	0.642 ± 0.072	0.926 ± 0.022	0.732 ± 0.078	0.604 ± 0.071
IBM (zero-shot)	Watson to IBM	0.842	0.589	0.853	0.612	0.522

These figures do not constitute external validation: the IBM records are wholly contained within the Watson training benchmark (Section 4.12), so both protocols evaluate the model on data it has already seen. Watson reference numbers (5-fold CV) are reproduced from Table 2.

### 4.13 SHAP stability and the risk of misinterpretation under correlated features

Two final concerns are addressed regarding the SHAP-based interpretation. *(i) Stability of the explanations*. Because ϕ_*j*_ is approximated through the DeepExplainer with a finite background set of 200 samples, the resulting attributions are random variables. To quantify their stability, we recompute the global ranking using 20 independent random seeds (each with an independently drawn background set and a reinitialized network retrained for 20 epochs from the early-stopping checkpoint), measuring the Spearman rank correlation of the top-20 feature ordering between each pair of runs. The mean rank correlation across the 202=190 pairwise comparisons is ρ = 0.94 ± 0.04, with all twenty runs agreeing on the identity of the top seven drivers (*OverTime, Environment Satisfaction, Marital Status (Single), Job Involvement, Years in Current Role, Age*, and *Distance From Home*); the per-feature mean absolute SHAP values are also reproducible, with a coefficient of variation below 7% for every top-20 feature. The global ranking and the directional patterns reported in Section 4.14 are therefore robust. *(ii) Correlated features*. A well-known limitation of any Shapley attribution computed with an interventional reference distribution is that it spreads the attribution across redundant features in proportion to their conditional independence; ignoring this can lead to interpretation pitfalls when two or more inputs encode overlapping information. Inspection of the Spearman correlation matrix on our 40 engineered features identifies three clusters with |ρ|>0.6: (*Age, Total Working Years, Years At Company*), (*Job Level, Monthly Income, Total Working Years*), and (*Marital Status (Single), Marital Status (Married)*). Within each cluster, individual SHAP values must be aggregated rather than read in isolation. For example, the joint contribution of the seniority-tenure cluster is best summarized by the sum ϕ_Age_+ϕ_TotalWorkingYears_+ϕ_YearsAtCompany_ = 1.40 on the margin scale, which exceeds any of the individual contributions and matches the canonical seniority effect documented in workforce-economics studies. To formalize this guidance, all interventions proposed in Section 5 are formulated at the cluster level (e.g., “support for young, low-tenure staff”) rather than at the level of any single correlated input, thereby avoiding the well-known pitfall of mistaking attribution dilution for low feature importance.

### 4.14 Global feature importance via SHAP

Having established that the deep network is competitive with strong baselines, we now interpret its decisions with SHAP on the margin (log-odds) scale. The global ranking of features by mean absolute SHAP value, restricted to the top 20 drivers, is shown as a horizontal bar chart in Figure 8 and with the corresponding distribution of directional effects as a beeswarm plot in Figure 9, whereas the detailed numerical values together with the underlying benchmark-level feature statistics are reported in Table 10. *OverTime* dominates the ranking with a mean absolute SHAP of 1.77, nearly twice the value of the next feature, indicating that overtime status is by far the strongest single driver of the model's predicted attrition log-odds on this benchmark. *Environment Satisfaction, Marital Status (Single), Job Involvement, Years in Current Role, Age*, and *Distance From Home* form the second tier, each with a mean absolute SHAP in the range 0.72–0.90; translated into the substantive vocabulary of the benchmark, this attribution profile assigns the largest predictive role to psychosocial-climate features (satisfaction and involvement), demographic features (single status, younger age) and structural features (tenure and commute), in close agreement with the qualitative drivers reported by the organizational-behavior literature on real workforces. The remaining top-20 features, spanning compensation (*Monthly Rate, Job Level*), career progression (*Years Since Last Promotion, Total Working Years*), and education (*Edu Field Medical/Life Sciences*), contribute at a substantially smaller scale but collectively explain a non-negligible fraction of the predicted log-odds. The beeswarm representation in Figure 9 adds the directional information: for every feature, red dots (high raw value) and blue dots (low raw value) separate cleanly on the two sides of the zero line, which means that the sign of each feature's effect on the predicted attrition log-odds is consistent across the benchmark and therefore yields a stable, audit-ready explanation profile.

**Table 10:** Top-20 drivers of the model's predicted attrition log-odds on the benchmark, ranked by mean absolute SHAP value ${\bar{\phi }}_{j}$ (log-odds scale), together with their benchmark-level descriptive statistics.

Rank	Feature	${\bar{\phi }}_{j}$	Mean	Std	Min	Max	Direction
1	OverTime	1.770	0.284	0.451	0	1	↑
2	Environment satisfaction	0.899	2.715	1.097	1	4	↓
3	Marital status (single)	0.866	0.311	0.463	0	1	↑
4	Job involvement	0.813	2.725	0.714	1	4	↓
5	Years in current role	0.766	4.265	3.626	0	18	↓
6	Age (years)	0.722	36.87	9.126	18	60	↓
7	Distance from home	0.722	9.222	8.156	1	29	↑
8	Num. companies worked	0.453	2.662	2.477	0	9	↑
9	Job level	0.446	2.067	1.113	1	5	↓
10	Job satisfaction	0.439	2.739	1.104	1	4	↓
11	Years since last promotion	0.411	2.200	3.229	0	15	↑
12	Work-life balance	0.377	2.766	0.702	1	4	↓
13	Total working years	0.376	11.34	7.833	0	40	↓
14	Monthly rate	0.323	14,287	7,137	2,094	26,999	↓
15	Relationship satisfaction	0.306	2.718	1.078	1	4	↓
16	Marital status (married)	0.303	0.464	0.499	0	1	↓
17	Travel (frequent)	0.292	0.191	0.393	0	1	↑
18	Edu field (medical)	0.284	0.313	0.464	0	1	↑
19	Sex (male)	0.262	0.595	0.491	0	1	—
20	Edu field (life sci.)	0.257	0.416	0.493	0	1	—

The “Direction” column reports the sign of the effect read from the beeswarm plot in Figure 9: ↑ (resp. ↓) denotes that a higher raw feature value increases (resp. decreases) the predicted attrition log-odds.

### 4.15 Non-linear effects and individual explanations

Beyond the global ranking, SHAP also provides a functional decomposition that captures the non-linear relationship between each feature and the model's predicted attrition log-odds. The dependence plots of the six most influential features, shown in Figure 7, reveal several interpretable patterns that could not be expressed by a linear coefficient: working overtime lifts the attrition log-odds by roughly +1.5 to +6 units, with the vast majority of records labeled as “overtime” clustered above +2; environment satisfaction shows a clearly monotone protective gradient, with a score of 1 pushing the log-odds up by as much as +3 and a score of 4 pulling it down by a similar amount; single marital status almost always contributes positively to attrition (+1 to +3), whereas marriage or divorce produces an essentially null or negative effect; job involvement, years in current role and age all exhibit strong negative gradients, with records corresponding to employees below roughly 30 years of age or in their first year in the current role concentrated in the high-risk half-plane, and older, longer-tenured records shifted toward substantial negative SHAP values. These non-linear thresholds constitute interpretable signatures of the model's decision logic on this benchmark and would, in any real-data follow-up, indicate the precise feature ranges in which targeted retention levers should be tested.

Complementary to the global view, Figures 10, 11 showcase two representative records with opposite predictions: a high-risk predicted-leaver record (#334, *f*(**x**) = +10.65, $\hat{P}\left(\text{Leave}\right)=1.000$) and a low-risk predicted-stayer record (#983, *f*(**x**) = −15.14, $\hat{P}\left(\text{Leave}\right)=0.000$). Both predictions are additively decomposed relative to the benchmark-mean baseline ϕ_0_ = *E*[*f*(**X**)] = −6.58 in agreement with the local-accuracy axiom of Shapley values. For record #334 (Figure 10), the leading positive contributions are *OverTime* (+3.57), low *Job Involvement* (+2.45), a long commute of 24 km (+1.59), *Marital Status (Single)* (+1.54), young age of 26 years (+1.37) and zero years in the current role (+1.02), whereas the only notable negative contribution comes from a high *Job Satisfaction* of 4 (−0.54); this configuration matches the archetypal “over-worked, under-engaged early-career” profile that the organizational-behavior literature on real workforces consistently identifies as high-risk for voluntary turnover. For record #983 (Figure 11), the same feature set works in the opposite direction: high *Job Involvement* of 4 (−1.67), older age of 55 (−1.51), long total working years of 31 (−1.11), no overtime (−0.94), short commute (−0.87), senior *Job Level* of 4 (−0.80) and strong *Work-Life Balance* of 4 (−0.66), while a mildly low *Environment Satisfaction* of 2 provides the only positive push (+0.64). Such case-level decompositions provide auditable, human-readable rationales that, in a real-data deployment, would convert the deep network into a transparent decision-support tool aligned with three candidate retention levers—overtime reduction, working-environment improvement, and commuting support—which we propose as data-driven hypotheses for managerial action rather than as validated interventions on this synthetic benchmark.

## 5 Conclusion

In this study, we developed an end-to-end SHAP-interpretable deep neural network for healthcare employee-attrition prediction on the publicly available Watson Healthcare synthetic benchmark of 1, 676 records (a healthcare-relabeled derivative of the IBM Watson HR Analytics dataset; see Section 3.2 for full provenance disclosure). The empirical evidence reported in Section 4 shows that the proposed compact MLP with a 40–128–64–32–2 funnel topology, trained on SMOTE-balanced data with AdamW, cosine annealing and early stopping, attains, under a leak-free cross-validation protocol, a 5-fold cross-validated AUC-ROC of 0.916 ± 0.030 and an AUC-PR of 0.713 ± 0.101, within one cross-fold standard deviation of—and not exceeding—logistic regression, random forest and gradient boosting (gradient boosting attains the highest mean AUC-ROC and AUC-PR; fold-level paired tests are mostly non-significant, while the pooled out-of-fold DeLong test finds the small ROC advantages of gradient boosting and logistic regression statistically significant; Section 4.5). Because the IBM HR Analytics dataset is the source from which the Watson benchmark was derived, it cannot serve as external validation (Section 4.12). The framework is robust to the choice of imbalance-handling strategy among SMOTE, ADASYN, Borderline-SMOTE and class-weighted cross-entropy (Section 4.10), and performs within one cross-fold standard deviation of two modern tabular deep architectures—NODE and FT-Transformer—while clearly outperforming TabNet at this sample size, all trained under the same leak-free protocol (Section 4.8). It is preferred over the gradient-boosting baseline not on raw accuracy grounds—on which gradient boosting holds a small, statistically significant ROC edge of 0.027 AUC-ROC points—but on the methodological grounds developed in Section 4.9, namely the smooth, differentiable attribution semantics of DeepSHAP, the well-calibrated log-odds outputs of the network, and the natural extensibility of the deep computational graph to longitudinal, multimodal real-data inputs; the analyses in Section 4.11 additionally confirm that the SMOTE step in our pipeline corrects rather than introduces majority-class bias, does not measurably degrade generalization on un-resampled evaluation folds, and does not compromise the reliability of SHAP-based explanations once the background reference is drawn from the un-resampled distribution. The subsequent SHAP analysis consistently identifies *OverTime, Environment Satisfaction, Marital Status (Single), Job Involvement, Years in Current Role, Age*, and *Distance From Home* as the seven dominant drivers of the model's predicted attrition log-odds on the benchmark, with interpretable non-linear thresholds and coherent individual-level waterfall decompositions. Beyond these quantitative findings, the principal value of the present study lies in unifying competitive predictive performance with dual-level, axiomatically grounded explainability within a single framework evaluated on a public healthcare-attrition benchmark: it turns an otherwise black-box deep network into an auditable, case-by-case analytical artifact whose decisions can be inspected at both global and local levels. It quantitatively characterizes the central role of job-satisfaction and compensation-related features—alongside workload and demographic context—in the model's attrition predictions, in a manner that closely echoes the qualitative drivers reported by the organizational-behavior literature on real workforces. It provides a transparent computational bridge between this benchmark and a set of candidate evidence-based interventions, by translating global and local SHAP insights into three concrete retention levers, namely overtime reduction, working-environment improvement, and commuting support, thereby offering a transferable template for interpretable workforce analytics in clinical settings. However, we emphasized that the present evidence is restricted to retrospective secondary analyses of two publicly available synthetic benchmarks. The proposed retention levers should therefore be regarded as data-driven hypotheses for managerial action rather than as clinically validated interventions, and prospective evaluation on real, properly de-identified hospital workforce data remains a necessary next step. We additionally acknowledge several explicit limitations that follow from the benchmark itself: (i) the records are synthetic and do not represent any real hospital workforce; (ii) the original IBM dataset is generic cross-sectoral HR data, and relabeling job roles obtain the healthcare framing, so the dataset cannot be used to make claims that are specific to nurses or allied-health professionals; (iii) a subset of the original outcome labels was modified by the dataset's contributor to facilitate machine-learning evaluation, implying that the absolute performance numbers reported here may not transfer one-for-one to real workforce data with intact label structure. These limitations directly motivate, but do not undermine, the methodological contribution of the present study, and they will guide the design of the prospective real-data follow-up study mentioned above; (iv) this study performs no economic or cost-effectiveness analysis (no cost, QALY, or incremental cost-effectiveness quantities are estimated), so the identified attrition drivers should be read only as candidate inputs that a subsequent, dedicated economic evaluation on real workforce data could price, and the contribution is methodological rather than health-economic. Building on this foundation, our future studies will extend the framework in several directions: scaling the pipeline to real, multi-center, and longitudinal hospital workforce datasets to jointly model time-varying covariates and survival-style attrition outcomes; integrating causal-inference techniques with Shapley attributions to move from correlational drivers toward intervention-ready, counterfactually valid retention levers; and enriching the feature space with unstructured signals, such as shift schedules, patient-care intensity indicators, and free-text engagement surveys, through multimodal representation learning; and deploying the model within real-world hospital human-resource information systems to enable prospective, closed-loop evaluation of the recommended interventions and, ultimately, a data-driven, explainable and clinically actionable pathway toward sustainable healthcare workforce retention.

## Data Availability

The dataset analyzed in this study is the publicly available Watson Healthcare Employee Attrition benchmark, distributed via the Kaggle data repository (contributor: J. P. Miller) and accessible at https://www.kaggle.com/datasets/jpmiller/employee-attrition-for-healthcare. We disclose the following provenance information in full transparency, in line with the description provided by the dataset's contributor on the Kaggle landing page: the data are synthetic and were not collected from any real hospital, healthcare system or workforce; the dataset is a relabeled derivative of the synthetic IBM Watson HR Analytics Employee Attrition & Performance dataset, in which the original generic job roles and departments were renamed to healthcare-domain values; and a subset of the original outcome labels was modified by the dataset's contributor to facilitate machine-learning evaluation. The dataset therefore contains no records of any real persons, no direct or indirect personal identifiers, and no real-world clinical or human-resource information. We use it strictly as a public, reproducible methodological benchmark for tabular attrition prediction and explainability research, and the numerical results reported in this paper should be read accordingly. The IBM HR Analytics Employee Attrition & Performance dataset (also synthetic, distributed publicly by IBM) is the source from which the Watson Healthcare benchmark was derived; it is referenced here only to document that provenance and was not used as an independent validation set. No new primary data were generated during the course of this study. The preprocessing pipeline, neural-network implementation and SHAP-based explanation code that support the findings reported in this paper are available from the corresponding author upon reasonable request.
