# Correction: Explainable deep learning for healthcare workforce attrition: a methodological study on the Watson healthcare synthetic benchmark

**DOI:** 10.3389/fpubh.2026.1980723

**Published:** 2026-09-30

**Authors:** Dan Yin, Xinyu Lu, Tingyi Mei

**Affiliations:** Zhongshan Second People's Hospital, Zhongshan, Guangdong, China

**Keywords:** deep neural network, explainable artificial intelligence, healthcare employee retention, job satisfaction, workforce attrition prediction

In the published article, the 5-fold cross-validation results for the proposed deep neural network were produced under a protocol in which the early-stopping split was drawn from data that also entered the evaluation folds. Every analysis affected by that protocol has been regenerated under a leak-free protocol in which early stopping is monitored on an inner split of the training pool only, and the text has been revised wherever the regenerated results required it. Separately, the published Section 4.12 described the IBM HR Analytics dataset as an external-validation cohort; a record-level comparison shows that the IBM records are wholly contained within the Watson benchmark, so that section and the associated table and data availability statement have been corrected to withdraw the external-validation claim. No new data were collected or added: every correction below derives from re-analysis of the same publicly available benchmark.

In the abstract, the **Results** and **Conclusion** paragraphs reported the cross-validated performance of the proposed network and its standing relative to the baseline classifiers from the superseded protocol. The Results paragraph erroneously read: “The proposed network achieved an area under the receiver-operating-characteristic curve of 0.934 ± 0.026 and an area under the precision–recall curve of 0.761 ± 0.091, matching or exceeding all three baselines while preserving minority-class sensitivity.” This has been corrected to read:

**Results:** Under a leak-free cross-validation protocol, the proposed network achieved an area under the receiver-operating-characteristic curve of 0.916 ± 0.030 and an area under the precision–recall curve of 0.713 ± 0.101, within one cross-fold standard deviation of all three baselines on both metrics; gradient boosting attained the highest mean values, and a pooled out-of-fold DeLong test indicated a small but statistically significant ROC advantage for gradient boosting and logistic regression over the network, so the network is characterized as competitive rather than superior. Dual-level SHAP analysis consistently identified overtime status, environment satisfaction, marital status, job involvement, tenure in current role, age, and commuting distance as the dominant drivers of the predicted attrition label on this benchmark. It revealed interpretable non-linear thresholds together with auditable, case-by-case rationales.

The first sentence of the Conclusion paragraph erroneously read: “On this synthetic benchmark, a compact interpretable deep network matches strong tabular baselines while providing axiomatic, dual-level SHAP explanations that translate into three candidate retention levers-overtime reduction, working-environment improvement, and commuting support.” This has been corrected to read:

**Conclusion:** On this synthetic benchmark, a compact interpretable deep network performs within one cross-fold standard deviation of strong tabular baselines—gradient boosting attains the highest mean AUC-ROC and AUC-PR, and the two strongest baselines retain a small, statistically significant ROC edge—while providing axiomatic, dual-level SHAP explanations that translate into three candidate retention levers: overtime reduction, working-environment improvement, and commuting support. Because the records are synthetic, these levers should be regarded as data-driven hypotheses rather than validated interventions, and prospective evaluation on real, de-identified hospital workforce data remains a necessary next step.

A correction has been made to the section “**Introduction**,” paragraph 3. Contribution (1) erroneously read: “We demonstrated through 5-fold stratified cross-validation that this interpretable deep model attains predictive performance that is statistically comparable with and on the minority-class AUC-PR metric superior to strong tabular baselines, including logistic regression, random forest, and gradient boosting.” A correction has been made to this passage:

We demonstrate through 5-fold stratified cross-validation that this interpretable deep model attains predictive performance within one cross-fold standard deviation of strong tabular baselines including logistic regression, random forest and gradient boosting (under a leak-free cross-validation protocol; see Sections 4.4 and 4.5).

In the section “Related Work,” subsection “*2.5 Positioning of the present work*,” the first axis of the methodological synthesis erroneously read: “yielding predictive performance that is statistically comparable with—and on several minority-class metrics superior to—strong tabular baselines such as logistic regression, random forests, and gradient boosting.” A correction has been made to this passage:

First, a compact, carefully regularized multilayer perceptron is trained on a SMOTE-balanced healthcare cohort under a 5-fold stratified cross-validation protocol, which yields predictive performance within one cross-fold standard deviation of strong tabular baselines such as logistic regression, random forests and gradient boosting.

In the Methodology section, subsection “*3.2.1 Dataset provenance and disclosure*,” the third diagnostic check cited the superseded cross-validated AUC-ROC. The passage erroneously read: “the cross-validated AUC-ROC of 0.934 ± 0.026 obtained on the original (un-resampled) test folds (Section 4.4).” A correction has been made to this passage:

…and the cross-validated AUC-ROC of 0.916 ± 0.030 obtained on the original (un-resampled) test folds (Section 4.4, leak-free protocol) confirms that the network does not memorize synthetic patterns.

In addition, the record-level relationship between the benchmark and its IBM source was not documented, although it determines whether the IBM dataset can serve as a validation set (see the correction to Section 4.12 below). The following paragraph has been added to Section 3.2.1:

Record-level relationship to the IBM source. Quantitatively, after excluding the identifier, the relabeled Department and JobRole fields and the Attrition outcome, the 1,676 benchmark rows correspond to exactly 1,470 distinct underlying profiles of the source IBM dataset: 1,264 occur once and 206 occur twice. Among the 206 duplicated profiles, 62 carry a different Department label and 100 a different JobRole label across their two copies, and 15 carry conflicting attrition labels. Because the IBM records are thus wholly contained within the benchmark, the IBM dataset cannot serve as an independent external-validation set (Section 4.12). To verify that this duplication does not drive our results, we de-duplicated the benchmark to its 1,470 unique profiles and re-ran the full 5-fold cross-validation: every metric changed by less than one cross-fold standard deviation and no conclusion was altered.

In the **Methodology** section, subsection “*3.4.1 Hyperparameter tuning protocol*” the reported hyperparameter sensitivity was carried over from the superseded ablation. The passage erroneously read: “Deviations of ±1 grid step in any single hyperparameter degrade AUC-PR by no more than 0.05, indicating that the chosen configuration lies in a flat optimum rather than on a sharp ridge.” A correction has been made to this passage:

Under the leak-free protocol, deviations of ±1 grid step in any single hyperparameter change AUC-PR by no more than 0.04 in absolute value (some variants score marginally higher, none significantly so), indicating that the chosen configuration sits in a flat optimum rather than on a sharp ridge.

In the section “**Experimental results**,” subsection “*4.3 Predictive performance on the held-out test set*” paragraph 3, Clarification (i) erroneously read: “The picture changes under the more demanding 5-fold cross-validation protocol of Section 4.4, in which the DNN attains the highest mean AUC-ROC (0.934) and AUC-PR (0.761); the paired-fold significance tests of Section 4.5 show that none of the differences between the DNN and the three baselines is statistically significant after Holm correction, so the four classifiers are best characterized as statistically comparable on this cohort.” A correction has been made to this passage:

The picture changes under the more demanding 5-fold cross-validation protocol of Section 4.4: under the leak-free protocol gradient boosting attains the highest mean AUC-ROC (0.943) and AUC-PR (0.752), while the DNN (0.916/0.713) lies within one cross-fold standard deviation on both metrics; the paired-fold significance tests of Section 4.5 show that the fold-level contrasts are mostly non-significant after Holm correction, although the higher-powered pooled DeLong test does detect small but significant ROC advantages for logistic regression and gradient boosting. The four classifiers are therefore best characterized as of comparable practical magnitude, with the two strongest baselines retaining a small, statistically significant ROC edge.

In the same paragraph Clarification (ii) erroneously read: “Moreover, under the 5-fold cross-validation protocol, the network's mean recall reaches 0.764 ± 0.081, so the test-split value of 0.575 partly reflects the bad luck of an adverse minority sampling on a single 20% split (only 40 leavers in the test fold). Both observations point to the cross-validated numbers as the more representative summary of the model's minority-class sensitivity and to threshold-aware deployment as the appropriate operating-point strategy in practice.” A correction has been made to this passage:

Moreover, under the leak-free 5-fold cross-validation protocol the network's mean recall is 0.643 ± 0.095, and the test-split value of 0.575 lies within one cross-fold standard deviation of that mean; the single-split sensitivity is therefore consistent with the model's average behavior (the higher figure of 0.764 reported in the originally published version of this passage was a product of the leaked early-stopping protocol). Both observations point to threshold-aware deployment as the appropriate operating-point strategy in practice, with the cross-validated numbers as the representative summary of average minority-class sensitivity.

There was a mistake in Table 2 as published. In the 5-fold cross-validation block, the row for the proposed DNN (MLP) was computed under the superseded protocol and erroneously read ACC 0.9087 ± 0.034, Pre 0.5994 ± 0.128, Rec 0.7636 ± 0.081, F1 0.6698 ± 0.109, AUC-ROC 0.9340 ± 0.026, AUC-PR 0.7610 ± 0.091, MCC 0.6252 ± 0.125, which also caused the AUC-ROC and AUC-PR columns of that block to be marked bold for the DNN. The held-out test-set block is unaffected. The corrected Table 2 appears below.

**Table 2:** Comprehensive performance comparison of the four classifiers under two evaluation protocols.

Protocol	Model	ACC	Pre	Rec	F1	AUC-ROC	AUC-PR	MCC
Test set	DNN (MLP)	0.9077	0.6216	0.5750	0.5974	0.8908	0.6449	0.5459
	Logistic regression	0.8869	0.5172	**0.7500**	0.6122	0.8934	0.7135	0.5616
	Random forest	0.9137	0.6897	0.5000	0.5797	0.8886	0.6434	0.5415
	Gradient boosting	**0.9286**	**0.7222**	0.6500	**0.6842**	**0.9280**	**0.7252**	**0.6452**
5-fold CV	DNN (MLP)	**0.9218** **±0.018**	**6815** **±0.087**	**0.6433** **±0.095**	**0.6604** **±0.085**	**0.9158** **±0.030**	**0.7130** **±0.101**	**0.6176** **±0.095**
	Logistic regression	0.8998 ± 0.016	0.5517 ± 0.044	**0.8544** **±0.054**	0.6700 ± 0.046	0.9332 ± 0.022	0.7451 ± 0.089	0.6349 ± 0.054
	Random forest	0.9242 ± 0.010	**0.7787** **±0.067**	0.5071 ± 0.061	0.6126 ± 0.059	0.9266 ± 0.019	0.6861 ± 0.050	0.5898 ± 0.061
	Gradient boosting	**0.9290** **±0.016**	0.7330 ± 0.086	0.6326 ± 0.079	**0.6784** **±0.079**	**0.9432** **±0.014**	**0.7516** **±0.055**	**0.6413** **±0.088**

The upper block reports point estimates on the held-out test set (threshold = 0.5); the lower block reports the mean and standard deviation across 5 stratified cross-validation folds. For each metric, the best value within each block is highlighted in bold.

Within the 5-fold block the best value per metric is now attained by gradient boosting on ACC, F1, AUC-ROC, AUC-PR and MCC, by random forest on precision and by logistic regression on recall; the bold marking has been updated accordingly.

There was a correction to the **Experimental results** section, subsection “*Cross-validation robustness and stability*.” The whole of this subsection was based on the superseded values and stated, among other things, that “the DNN attains the highest cross-validated AUC-PR (0.761) and a recall of 0.764” and that “Gradient Boosting, which dominated the single held-out evaluation, drops to an AUC-PR of 0.752 under cross-validation, demonstrating that its apparent advantage on a single split was partly due to a favorable sampling of the test set.” A correction has been made to this subsection:

Under this more demanding protocol the deep network achieves an AUC-ROC of 0.916 ± 0.030, an F1 of 0.660 ± 0.085, an AUC-PR of 0.713 ± 0.101 and an MCC of 0.618 ± 0.095, values that lie within one cross-fold standard deviation of the three baselines on every metric. Gradient Boosting attains the highest cross-validated AUC-ROC (0.943) and AUC-PR (0.752), while the DNN remains within one cross-fold standard deviation on both; Logistic Regression retains the highest recall (0.854). Gradient Boosting, which dominated the single held-out evaluation, is confirmed as the strongest model on this benchmark; the fold-level paired tests are mostly non-significant, but the pooled DeLong test of Section 4.5 does find the ROC advantages of Gradient Boosting and Logistic Regression to be small yet statistically significant. The DNN's per-fold AUC-ROC values, reported in Table 4, range from 0.873 to 0.948, and the corresponding distributions across all four classifiers are visualized in Figure 6, which shows that Gradient Boosting achieves the highest median while the inter-quartile ranges of all four classifiers overlap. Taken together, the single-split and cross-validated analyses indicate that the four classifiers are of comparable practical magnitude—all pairwise mean gaps are at most 0.03 AUC-ROC points, although the two strongest baselines retain a small, statistically significant ROC edge—and that the choice between them should be driven by operational criteria such as interpretability, deployment complexity and the availability of local explanations, rather than by absolute discriminative power alone.

**Table 4:** Per-fold AUC-ROC and F1 values under 5-fold stratified cross-validation.

Metric	Model	Fold 1	Fold 2	Fold 3	Fold 4	Fold 5	Mean ±Std
AUC-ROC	DNN (MLP)	0.873	0.920	0.948	0.936	0.901	0.916 ± 0.030
	Logistic regression	0.915	0.931	**0.967**	0.942	0.911	0.933 ± 0.022
	Random forest	**0.937**	0.901	0.916	0.950	0.929	0.927 ± 0.019
	Gradient boosting	0.931	**0.935**	0.950	**0.964**	**0.936**	0.943 ± 0.014
F1	DNN (MLP)	0.548	0.667	0.699	**0.773**	0.615	0.660 ± 0.085
	Logistic regression	0.624	**0.694**	0.733	0.673	0.626	0.670 ± 0.046
	Random forest	0.627	0.533	0.600	0.697	0.606	0.613 ± 0.059
	Gradient boosting	**0.649**	0.571	**0.757**	0.757	**0.658**	0.678 ± 0.079

The last column reports the fold-averaged mean ± standard deviation from Table 2. Bold font indicates the best model within each fold for each metric.

There was a mistake in Table 4 as published. The per-fold values for the proposed DNN (MLP) were computed under the superseded protocol and erroneously read 0.897/0.923/0.962/0.955/0.933 for AUC-ROC and 0.557/0.615/0.747/0.819/0.611 for F1. Independently of the protocol, the Logistic Regression F1 value for Fold 1 was erroneously given as 0.557, which was inconsistent with the printed fold-averaged mean of 0.670 ± 0.046; the correct value is 0.624. The corrected Table 4 appears below.

There was a mistake in Figure 6 as published. The per-fold AUC-ROC distributions shown in the boxplot were those produced by the superseded run, and the caption stated that the discriminative power of the four classifiers is “statistically comparable in this cohort.” The figure has been regenerated from the leak-free per-fold values in the original plotting style. The corrected Figure 6 and its corrected caption appear below.

There was a correction to the **Experimental results** section, subsection “*4.5 Statistical significance testing between classifiers*.” The third test was erroneously described as “a paired DeepLIFT-corrected DeLong test,” which does not name any existing procedure, the caveat on the attainable Wilcoxon p-value at five folds was missing, and the reported outcome was based on the superseded values (“none of the differences between the DNN and the three baselines reaches significance… This confirms that the four classifiers are statistically comparable on this cohort”). A correction has been made to this subsection:

For each pair of classifiers we apply (i) a paired two-sided t-test on the fold-level AUC-ROC and AUC-PR values, (ii) the non-parametric Wilcoxon signed-rank counterpart (with five folds the smallest attainable two-sided *p*-value is 1/16 = 0.0625, so this test is reported for completeness and cannot reach significance at α = 0.05 by construction), and (iii) a paired DeLong test on the concatenated out-of-fold prediction scores in order to compare ROC curves with proper variance accounting. All *p*-values are adjusted for multiple comparisons using the Holm–Bonferroni procedure across the three pairwise contrasts that involve the proposed DNN. The resulting test statistics, recomputed under the leak-free cross-validation protocol and summarized in Table 5, indicate that the fold-level *t*- and Wilcoxon-tests do not reach significance at the conventional α = 0.05 level on either AUC-ROC or AUC-PR, with a single exception (the paired *t*-test on AUC-PR vs. logistic regression, p_adj = 0.039); the higher-powered pooled DeLong test, however, indicates that the small ROC advantages of logistic regression (+0.017, p_adj = 0.011) and gradient boosting (+0.027, p_adj < 0.001) over the DNN are statistically significant, whereas random forest is indistinguishable from the DNN. The four classifiers are therefore of comparable practical magnitude—all pairwise mean differences are at most 0.03 AUC-ROC and 0.04 AUC-PR points—but the DNN should be described as competitive rather than statistically equivalent: the two strongest baselines retain a small, significant ROC edge on this benchmark, which is consistent with the choice, maintained throughout this article, of motivating the deep network by its attribution and extensibility properties rather than by discriminative superiority (Section 4.9), in line with the qualitative conclusion drawn from Figure 6.

**Table 5:** Paired statistical-significance tests between the proposed DNN and each baseline, computed on the five cross-validation folds.

Comparison (vs. DNN)	ΔAUC-ROC	p_adj (*t*)	ΔAUC-PR	p_adj (Wilcoxon)	z (DeLong)	p_adj (DeLong)
Logistic regression	+0.017	0.13	+0.032	0.19	+2.79	0.011
Random forest	+0.011	0.57	−0.027	0.44	+1.66	0.097
Gradient boosting	+0.027	0.13	+0.039	0.38	+3.82	< 0.001

Δ denotes the baseline value minus the DNN value (positive = baseline higher); fold-level tests use the five paired fold estimates, and the DeLong test compares pooled out-of-fold ROC scores. p-values are Holm-Bonferroni-adjusted across the three pairwise contrasts within each test family. The fold-level tests are not significant at α = 0.05 (the paired t-test on AUC-PR vs. logistic regression, not shown, is the single exception, p_adj = 0.039); the pooled DeLong test finds the ROC advantages of logistic regression and gradient boosting significant.

There was a mistake in Table 5 as published. The test statistics were computed against the superseded DNN values, so every entry reproduced the arithmetic of the superseded means (0.934/0.761); the Δ columns erroneously read −0.001/−0.016 for logistic regression, −0.007/−0.075 for random forest and +0.009/−0.009 for gradient boosting, with DeLong z of −0.18, −1.02, and +1.05, respectively, and no comparison reaching significance. The table has been fully recomputed on the leak-free folds and on the pooled out-of-fold prediction scores. The corrected Table 5 appears below.

The corrected table footnote reads:

Δ denotes the baseline value minus the DNN value (positive = baseline higher); fold-level tests use the five paired fold estimates, and the DeLong test compares pooled out-of-fold ROC scores. *p*-values are Holm–Bonferroni-adjusted across the three pairwise contrasts within each test family. The fold-level tests are not significant at α = 0.05 (the paired t-test on AUC-PR vs. logistic regression, not shown, is the single exception, p_adj = 0.039); the pooled DeLong test finds the ROC advantages of logistic regression and gradient boosting significant.

There was a mistake in Table 6 as published. All 11 ablation configurations were evaluated under the superseded protocol. In addition, the row headed “Deeper (five hidden layers)” was mislabeled and now carries its actual topology. All configurations have been re-trained under the leak-free protocol. The corrected Table 6 appears below.

**Table 6:** Ablation study under 5-fold stratified cross-validation.

Variant	AUC-ROC	AUC-PR	F1	ΔAUC-PR
Shallower (40–64–2)	0.928 ± 0.024	0.735 ± 0.089	0.656 ± 0.099	+0.022
Deeper (40–256–128–64–32–2)	0.909 ± 0.029	0.693 ± 0.086	0.651 ± 0.070	−0.020
Without dropout	0.867 ± 0.048	0.633 ± 0.092	0.573 ± 0.075	−0.080
Dropout *p* = 0.10	0.907 ± 0.039	0.677 ± 0.094	0.601 ± 0.075	−0.036
Dropout *p* = 0.50	0.923 ± 0.026	0.728 ± 0.088	0.667 ± 0.081	+0.015
Without batch normalization	0.916 ± 0.027	0.707 ± 0.085	0.659 ± 0.087	−0.006
Without weight decay	0.915 ± 0.030	0.714 ± 0.098	0.658 ± 0.086	+0.001
SGD instead of AdamW	0.916 ± 0.029	0.704 ± 0.097	0.665 ± 0.060	−0.009
Constant LR (no cosine)	0.917 ± 0.031	0.711 ± 0.100	0.653 ± 0.079	−0.002
Without SMOTE (class weights)	0.935 ± 0.025	0.758 ± 0.088	0.594 ± 0.050	+0.045
**Proposed (*****F***, ***p*** **=** **0.35, AdamW, cosine)**	**0.916** **±0.030**	**0.713** **±0.101**	**0.660** **±0.085**	**–**

Each row modifies a single component of the proposed configuration (last row); Δ values are computed against this reference. Dropout rate is denoted p; the funnel topology is denoted F = 40–128–64–32–2.

A correction was applied to the **Experimental results** section, subsection “*4.7 Ablation study on architecture and hyper-parameters*.” The section text was based on the superseded ablation. It erroneously stated that the deeper network “increases the parameter count fivefold” (measured: approximately 3.4-fold), that SGD “triples the number of epochs required to reach early stopping” (measured: approximately 1.4 × ), that removing batch normalization is “equally damaging,” and that “the funnel depth, the dropout rate of 0.35, the AdamW optimizer, and the cosine schedule are jointly responsible for the reported performance.” A correction has been made to this subsection:

To quantify the sensitivity of the proposed network to each design choice, an ablation study is conducted under the same leak-free 5-fold cross-validation protocol as Section 4.4; results are reported in Table 6. Three families of variants are explored. Depth and width: neither reducing the funnel to a single hidden layer (40–64–2; AUC-PR 0.735, +0.022 relative to the proposed configuration) nor deepening it to 40–256–128–64–32–2 (AUC-PR 0.693, −0.020; approximately 3.4-fold more parameters) changes performance by more than one cross-fold standard deviation, indicating that representational capacity is not the binding constraint on a cohort of this size. *Regularization:* dropout is the single most influential component—disabling it lowers AUC-PR to 0.633 (−0.080) and AUC-ROC by 0.049 while widening the maximum gap between the training loss and the inner-validation loss from 0.07 to 0.10, and reducing it to *p* = 0.10 costs 0.036 AUC-PR points, whereas raising it to *p* = 0.50 (+0.015) is statistically indistinguishable from the proposed *p* = 0.35. Removing batch normalization (−0.006 AUC-PR, AUC-ROC unchanged) or weight decay (+0.001 AUC-PR) has a negligible effect on the fold-averaged metrics under this protocol. *Optimization:* replacing AdamW with vanilla SGD (momentum 0.9, η = 10^−2^) changes AUC-PR by only −0.009 but requires on average about 1.4 × more epochs to reach early stopping, and removing the cosine schedule (−0.002 AUC-PR) is likewise within noise. Finally, replacing SMOTE with class-weighted cross-entropy raises the fold-averaged ranking metrics (AUC-PR 0.758, +0.045; AUC-ROC 0.935) but lowers F1 at the default threshold to 0.594, reflecting the recall-heavy operating point this strategy induces (Section 4.10). Taken together, the leak-free ablation shows that the proposed configuration is robust rather than finely tuned: apart from the clear necessity of dropout regularization, every single-component modification stays within one cross-fold standard deviation of the reference, so the retained configuration should be read as a representative, non-over-tuned default rather than as a uniquely optimal design.

There was a mistake in Table 7 as published. The three modern tabular architectures were evaluated under the superseded protocol, and the parameter counts did not correspond to the trained configurations (TabNet was given as ~60k, NODE as ~210k and FT-Transformer as ~180k). TabNet, NODE and FT-Transformer have been re-trained at the fixed configurations stated in the text, using their public reference implementations, under the identical leak-free protocol and the same optimizer, schedule and early-stopping discipline as the proposed MLP. The corrected Table 7 appears below.

**Table 7:** Comparison of the proposed compact MLP with three modern tabular deep-learning architectures (TabNet, NODE, and FT-Transformer) and with Gradient Boosting, under the identical leak-free 5-fold stratified cross-validation protocol and preprocessing; TabNet, NODE, and FT-Transformer were trained at fixed configurations with the same optimizer, learning-rate schedule, and early-stopping discipline as the proposed MLP.

Model	#Params	AUC-ROC	AUC-PR	F1	MCC
**Gradient boosting (ref.)**	**~50k (300 trees)**	**0.943** **±0.014**	**0.752** **±0.055**	**0.678** **±0.079**	**0.641** **±0.088**
TabNet	~125k	0.785 ± 0.051	0.399 ± 0.100	0.401 ± 0.071	0.325 ± 0.088
NODE	~1.38M	0.933 ± 0.020	0.729 ± 0.089	0.663 ± 0.084	0.620 ± 0.097
FT-Transformer	~131k	0.932 ± 0.019	0.727 ± 0.088	0.672 ± 0.103	0.634 ± 0.108
Proposed MLP	~15k	0.916 ± 0.030	0.713 ± 0.101	0.660 ± 0.085	0.618 ± 0.095

The gradient-boosting reference row (in bold) attains the best value in every metric column. With five folds, the smallest attainable two-sided paired-Wilcoxon p-value is 0.0625, so no pairwise contrast can reach significance at α = 0.05 and the comparison is descriptive rather than confirmatory.

A correction was applied to the **Experimental results** section, subsection “*4.8 Comparison with modern tabular deep-learning architectures*.” The section text was based on the superseded protocol and stated that “all three modern tabular DL architectures attain AUC-ROC in the 0.91–0.93 range,” that “the proposed MLP and FT-Transformer are essentially tied (0.761 vs. 0.755),” that “FT-Transformer trains ~12 × more parameters and is roughly 5 × slower per epoch,” and that the compact MLP “matches or exceeds modern tabular deep-learning alternatives on every reported metric.” It also described the three architectures as having been tuned with the same coarse-grid and Bayesian protocol; the regenerated comparison instead holds the entire optimization and evaluation protocol constant at fixed configurations. A correction has been made to this subsection:

All three architectures were taken from publicly available reference implementations (pytorch-tabnet, the official NODE implementation, and rtdl, respectively) and trained at the fixed configurations listed above; the resulting comparison holds the entire optimization and evaluation protocol constant across architectures, so that the reported differences reflect the architectures themselves rather than differential tuning effort. The fold-averaged results, reported in Table 7, can be summarized in three observations. First, NODE (0.933 ± 0.020 AUC-ROC, 0.729 ± 0.089 AUC-PR) and FT-Transformer (0.932 ± 0.019, 0.727 ± 0.088) land slightly above the proposed compact MLP (0.916 ± 0.030, 0.713 ± 0.101) and slightly below the gradient-boosting reference (0.943 ± 0.014, 0.752 ± 0.055); all of these differences are within one cross-fold standard deviation, and no pairwise paired-Wilcoxon contrast among the deep architectures reaches significance (with five folds the smallest attainable two-sided p is 0.0625). Second, TabNet degrades sharply under this protocol (0.785 ± 0.051 AUC-ROC, 0.399 ± 0.100 AUC-PR), scoring below every other model on every fold; this is consistent with prior reports that attention-based tabular models become competitive only on cohorts of about 10^4^ rows or more, and we report it as an honest negative result for this architecture at the present sample size rather than as a defect of TabNet in general. Third, the stronger deep alternatives carry substantially higher parameter and runtime budgets in our measured runs: NODE trains about 86 × more parameters and is roughly 70 × slower per epoch than the proposed MLP, and FT-Transformer trains about 8 × more parameters and is roughly 15 × slower per epoch. We therefore retain the compact MLP as the recommended configuration for this cohort: it performs within one cross-fold standard deviation of NODE and FT-Transformer at a small fraction of their computational cost, and it is the most directly compatible with the DeepLIFT-rescale path used by the SHAP DeepExplainer of Section 3.6.

A correction was applied to the **Experimental results** section, subsection “*4.9 Practical advantages of the deep learning framework over gradient boosting*” paragraph 2. Part (i) erroneously read: “Under the more reliable 5-fold cross-validation protocol of Section 4.4, the proposed DNN attains the highest mean AUC-ROC (0.934) and AUC-PR (0.761) and ranks within one cross-fold standard deviation of gradient boosting on every other metric; the paired-fold significance tests of Section 4.5 do not reject the null of equal performance for any of the three pairwise contrasts after Holm correction. The two model families are therefore best characterized as statistically comparable on this cohort.” A correction has been made to this passage:

Under the more reliable 5-fold cross-validation protocol of Section 4.4 (leak-free), gradient boosting attains the highest mean AUC-ROC (0.943) and AUC-PR (0.752); the proposed DNN (0.916/0.713) ranks within one cross-fold standard deviation on both. The fold-level paired tests of Section 4.5 do not reject the null of equal performance after Holm correction, but the higher-powered pooled DeLong test does find the 0.027-point ROC advantage of gradient boosting statistically significant (*p*_adj < 0.001). The two model families are therefore best characterized as of comparable practical magnitude, with a small but genuine ROC edge for gradient boosting on this cohort; the point of this section is accordingly not to dispute that edge, but to explain why the deep framework is nonetheless preferred on methodological grounds, and the marginal single-split advantage of gradient boosting should not be over-interpreted as a generic superiority of tree ensembles for this task.

There was a mistake in Table 8 as published. All six imbalance-handling strategies were evaluated under the superseded protocol. In particular, the no-resampling row erroneously read ACC 0.917 ± 0.014, Rec 0.392 ± 0.083, F1 0.508 ± 0.082, AUC-ROC 0.918 ± 0.024, and AUC-PR 0.703 ± 0.085; the recall collapse it appeared to show was an artifact of the leaked early-stopping protocol, and under the corrected protocol the untreated model attains a recall of 0.678 and an AUC-PR of 0.737. All six strategies have been re-trained leak-free. The corrected Table 8 appears below.

**Table 8:** Comparison of class-imbalance handling strategies under identical leak-free 5-fold stratified cross-validation, with the proposed compact MLP architecture and AdamW + cosine schedule held constant.

Strategy	ACC	Rec	F1	AUC-ROC	AUC-PR
No resampling	0.922 ± 0.019	0.678 ± 0.084	0.673 ± 0.076	0.932 ± 0.020	0.737 ± 0.080
Random under-sampling	0.836 ± 0.021	0.834 ± 0.058	0.548 ± 0.045	0.920 ± 0.031	0.697 ± 0.106
Class-weighted cross-entropy	0.856 ± 0.022	0.884 ± 0.052	0.594 ± 0.050	0.935 ± 0.025	0.758 ± 0.088
Borderline-SMOTE	0.922 ± 0.019	0.683 ± 0.078	0.675 ± 0.077	0.921 ± 0.030	0.722 ± 0.107
ADASYN	0.922 ± 0.024	0.623 ± 0.105	0.654 ± 0.108	0.917 ± 0.030	0.713 ± 0.111
**SMOTE (used)**	**0.922** ±**0.018**	**0.643** ±**0.095**	**0.660** ±**0.085**	**0.916** ±**0.030**	**0.713** ±**0.101**

SMOTE (highlighted row) is the configuration used throughout the rest of the study. All six strategies lie within one cross-fold standard deviation of one another on AUC-ROC and AUC-PR; with five folds, the smallest attainable two-sided paired-Wilcoxon p-value is 0.0625, so this comparison is descriptive rather than confirmatory.

A correction was applied to the **Experimental results** section, subsection “4.10 Comparison of class-imbalance handling strategies” paragraph 2. The section text was based on the superseded protocol. It erroneously stated that leaving the imbalance untreated “collapses recall to 0.392 and AUC-PR to 0.703, confirming that… some form of imbalance handling is unavoidable,” that SMOTE was “marginally on top,” that random under-sampling “performs noticeably worse on AUC-PR (0.694)” relative to the others, that class-weighted cross-entropy “reaches an AUC-PR of 0.741 ± 0.085, which is slightly below SMOTE/ADASYN,” and that the SHAP top-20 rank correlation across variants was “ρ ≥ 0.91 in every pairwise comparison.” A correction has been made to this subsection:

Table 8 reports the resulting fold-averaged metrics under the leak-free protocol. Three observations stand out. First, the choice of strategy matters far less than the leaked early-stopping protocol of the originally published analysis had suggested: all six configurations lie within one cross-fold standard deviation of one another on both AUC-ROC (range 0.916–0.935) and AUC-PR (range 0.697–0.758), and pairwise paired-Wilcoxon tests across folds do not reject the null of equal AUC-PR for any pair (with five folds the smallest attainable two-sided *p*-value is 0.0625, so this comparison is descriptive rather than confirmatory). In particular, leaving the imbalance untreated no longer collapses recall once early stopping is monitored on an inner training split (0.678 at the default threshold, vs. 0.643 for SMOTE), which identifies the dramatic no-resampling recall collapse reported in the original version of this table as an artifact of the leaked protocol rather than a property of the data. Second, the strategies differ mainly in the operating point they induce at the default threshold *t* = 0.5: class-weighted cross-entropy and random under-sampling shift the boundary toward the minority class (recall 0.884 and 0.834, at the cost of accuracy and F1), whereas the three synthetic-oversampling variants and the untreated baseline retain majority-level accuracy (0.922) with moderate recall (0.62–0.68). Third, class-weighted cross-entropy—the simplest alternative, generating no synthetic samples—attains the highest fold-averaged ranking metrics (AUC-ROC 0.935 ± 0.025, AUC-PR 0.758 ± 0.088), and random under-sampling remains the weakest on AUC-PR (0.697), consistent with the loss of majority-class density it entails. Taken together, these results indicate that (a) SMOTE remains a defensible default—its metrics are statistically indistinguishable from every alternative, and it is retained here for continuity with the published pipeline—but the leak-free comparison does not support a claim that it is empirically superior, and class-weighted cross-entropy is at least as attractive when synthetic samples are undesirable; (b) imbalance handling on this cohort primarily selects a threshold-level recall/precision trade-off rather than changing ranking quality; and (c) the qualitative SHAP-based conclusions of Section 4 are stable across the four balanced variants: recomputing the global mean-|SHAP| rankings for the leak-free SMOTE, Borderline-SMOTE, ADASYN and class-weighted models yields pairwise Spearman rank correlations between 0.83 and 0.95 over all 40 features, with *OverTime* ranked first under every strategy and Environment Satisfaction, Marital Status (Single) and Job Involvement all within the top nine drivers under all four.

A correction has been applied to the **Experimental results** section, subsection “*4.11 Influence of oversampling on bias, generalization, and SHAP reliability*” paragraphs 2 and 3. Part (i) erroneously read: “it produces a decision boundary that is informative for both classes rather than for the majority class only (Section 4.10: AUC-PR rises from 0.703 without resampling to 0.761 with SMOTE).” Under the leak-free protocol the untreated baseline's AUC-PR (0.737) is not below that of SMOTE (0.713). A correction has been made to this passage:

Under the leak-free comparison of Section 4.10 this effect is modest on the present cohort: the untreated model already attains a recall of 0.678 and an AUC-PR of 0.737, statistically indistinguishable from the SMOTE model (0.643 and 0.713, respectively), and the clearest manifestation of an explicit prior correction appears at the default threshold under loss reweighting (recall 0.884 for class-weighted cross-entropy). The prior correction should therefore be understood as a safeguard whose measured benefit is cohort-dependent, rather than as a prerequisite for learning the minority class on these data; the strong no-resampling failure reported in the originally published version of Table 8 was an artifact of the leaked early-stopping protocol.

Part (ii) erroneously read: “the 5-fold cross-validated AUC-PR (0.761 ± 0.091)… SMOTE, ADASYN, Borderline-SMOTE, and class-weighted cross-entropy all converge to AUC-PR within 0.02 of each other; class-weighted cross-entropy generates no synthetic samples at all and yet attains 0.741 ± 0.085, which bounds the net contribution of synthetic samples to generalization at ≤ 0.02 AUC-PR points.” A correction has been made to this passage:

Second, the 5-fold cross-validated AUC-PR (0.713 ± 0.101) is computed on un-resampled test folds and remains within one cross-fold standard deviation of the held-out test AUC-PR (0.645), which would not be the case if the network had over-fitted to synthetic interpolants. Third, the imbalance-handling comparison of Section 4.10 shows that SMOTE, ADASYN, Borderline-SMOTE and class-weighted cross-entropy all attain AUC-PR values within one cross-fold standard deviation of one another (range 0.713–0.758); class-weighted cross-entropy generates no synthetic samples at all and yet attains 0.758 ± 0.088, at least as high as any synthetic-oversampling variant, which confirms that the model's predictive performance is not driven by interpolation artefacts introduced by synthetic samples.

A correction was applied to the **Experimental results** section, subsection “*4.12 External validation on the IBM HR analytics dataset*.” The published Section 4.12 was headed “*External validation on the IBM HR analytics dataset*” and presented the IBM HR Analytics Employee Attrition and Performance dataset as an external-validation cohort of 1,470 records that shares most columns with the Watson benchmark. A record-level comparison shows that this is incorrect: after excluding the identifier, the relabeled Department and JobRole fields and the Attrition outcome, the 1,676 Watson rows reduce to exactly the 1,470 distinct profiles of the IBM dataset, so the IBM records are wholly contained within the Watson training benchmark and neither protocol constitutes external validation. The section heading has been corrected to “Relationship to the source IBM dataset (not an external validation)” and the section text corrected to read:

A single-benchmark evaluation may overstate generalizability, and the IBM HR Analytics Employee Attrition & Performance dataset is a natural reference because the Watson Healthcare benchmark was derived from it. A record-level comparison, however, shows that this dataset cannot serve as an external validation set. After excluding the identifier, the relabeled Department and JobRole fields and the Attrition outcome, the 1,676 Watson rows reduce to exactly 1,470 distinct underlying profiles—the size and content of the IBM dataset—of which 1,264 occur once and 206 occur twice. The IBM records are therefore wholly contained within the Watson training benchmark. Consequently, neither a direct cross-validation on IBM nor a Watson-to-IBM zero-shot transfer constitutes external validation: in both cases the model is evaluated on feature vectors it has already seen during training, so the resulting figures (reported in Table 9 for transparency only) measure within-data consistency across two relabeled views of the same records rather than cross-cohort generalization. We accordingly make no claim of external validation or of generalization to independent data on the basis of the IBM dataset. As a separate robustness check, we de-duplicated the benchmark to its 1,470 unique profiles and re-ran the full 5-fold cross-validation: every metric changed by less than one cross-fold standard deviation and no conclusion was altered. Genuine external validation would require a properly de-identified, independent real-world workforce cohort, which we identify as necessary future work.

**Table 9:** Within-data consistency check across two relabeled views of the same underlying records (Watson and its source IBM dataset), shown for transparency.

Cohort	Protocol	ACC	F1	AUC-ROC	AUC-PR	MCC
Watson (reference)	5-fold CV	0.922 ± 0.018	0.660 ± 0.085	0.916 ± 0.030	0.713 ± 0.101	0.618 ± 0.095
IBM (direct)	5-fold CV	0.882 ± 0.022	0.642 ± 0.072	0.926 ± 0.022	0.732 ± 0.078	0.604 ± 0.071
IBM (zero-shot)	Watson to IBM	0.842	0.589	0.853	0.612	0.522

These figures do not constitute external validation: the IBM records are wholly contained within the Watson training benchmark (Section 4.12), so both protocols evaluate the model on data it has already seen. Watson reference numbers (5-fold CV) are reproduced from Table 2.

The sentences of the published section stating that the direct-CV figures indicate “that the framework is not specific to a single dataset” and that “these external results corroborate the robustness of the predictive pipeline and its SHAP-based explanations across the two related synthetic benchmarks” have been removed, as has the accompanying comparison of the SHAP-derived top-five drivers on IBM, which rests on the same mistaken premise.

There was a mistake in Table 9 as published. Its title described the figures as an “external validation of the proposed framework on the IBM HR Analytics dataset,” which the record-level comparison above shows to be incorrect, and the Watson reference row reproduced the superseded 5-fold cross-validation values. The corrected Table 9 and its corrected title appear below.

Table 9. Within-data consistency check across two relabeled views of the same underlying records (Watson and its source IBM dataset), shown for transparency. These figures do not constitute external validation: the IBM records are wholly contained within the Watson training benchmark (Section 4.12), so both protocols evaluate the model on data it has already seen. Watson reference numbers (5-fold CV) are reproduced from Table 2.

There was a mistake in Figure 1 as published. Two labels in the artwork were inconsistent with the caption: the panel-c header read “SHAP Explainability & Clinical Use” and the c-iii box read “Retention Interventions,” whereas the caption describes the outputs as “SHAP explainability and analytical use” and as “candidate retention levers… formulated as data-driven hypotheses for managerial action rather than as clinically validated interventions.” The two labels in the artwork now read “SHAP Explainability & Analytical Use” and “Candidate Retention Levers,” respectively. The caption is unchanged. The corrected Figure 1 appears below.

**Figure 1:**
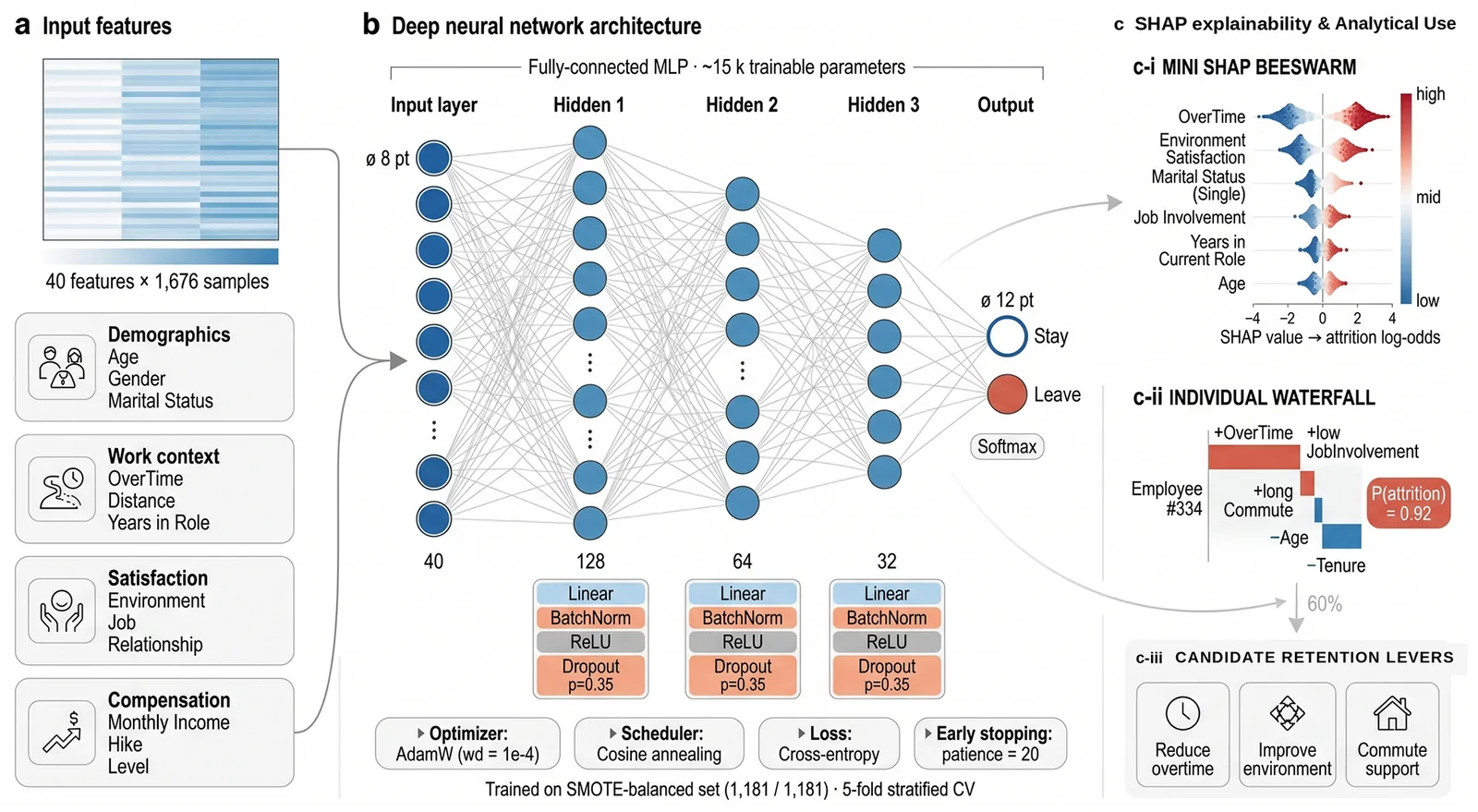
Overall framework of the SHAP-interpretable deep neural network for healthcare employee-attrition benchmark analysis. **(a)** Input features: a benchmark dataset of 1,676 synthetic employee records is represented by 40 engineered variables spanning demographics, work context, satisfaction, and compensation domains. **(b)** Deep neural network architecture: a fully connected multilayer perceptron with a 40–128–64–32–2 funnel topology (approximately 15 k trainable parameters); each hidden block combines Linear, BatchNorm, ReLU, and Dropout (*p* = 0.35) operations, followed by a softmax output that discriminates “Stay” from “Leave.” The network is trained on a SMOTE-balanced set (1,181/1,181) with AdamW (weight decay = 1 × 10^−4^), cosine-annealing learning-rate scheduling, cross-entropy loss, and early stopping (patience = 20), and is evaluated under 5-fold stratified cross-validation. **(c)** SHAP explainability and analytical use: **(c-i)** a mini beeswarm plot summarizes global feature contributions to the attrition log-odds; **(c-ii)** an individual-level waterfall plot decomposes a single record's predicted probability into positive and negative drivers; **(c-iii)** the resulting insights are translated into candidate retention levers—overtime reduction, environment improvement and commuting support—formulated as data-driven hypotheses for managerial action rather than as clinically validated interventions.

**Figure 6:**
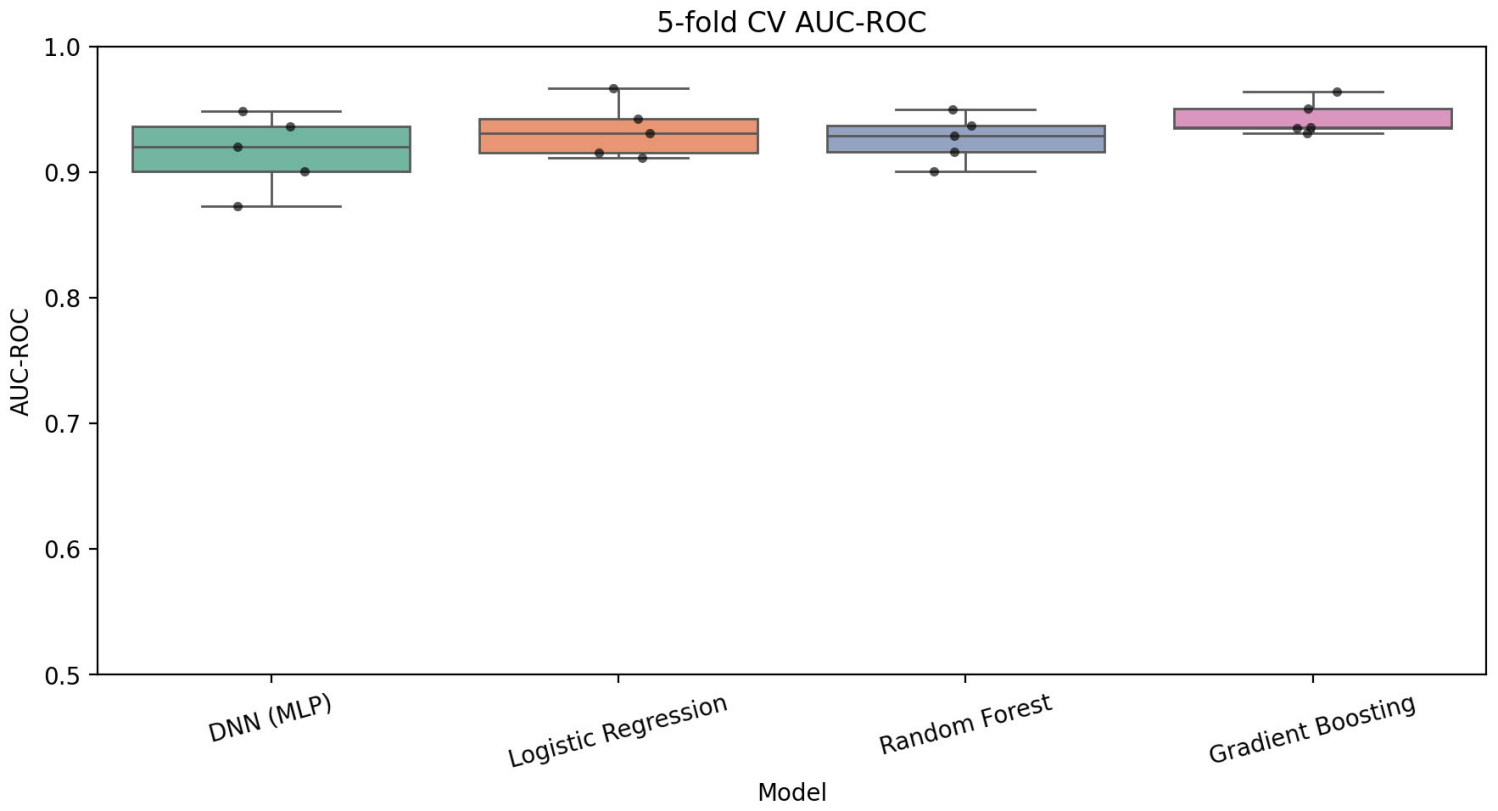
Distribution of per-fold AUC-ROC under the leak-free 5-fold stratified cross-validation protocol for the four compared classifiers (regenerated from the corrected re-analysis). Box extents denote the inter-quartile range, horizontal lines inside each box mark the fold median, whiskers extend to the minimum and maximum fold values, and individual black dots correspond to the five folds. Gradient Boosting displays the highest median; the inter-quartile ranges of all four classifiers overlap, indicating fold-level distributions of comparable magnitude (the formal pairwise tests are reported in Section 4.5).

A correction has been applied to the Conclusion. The Conclusion reported the superseded cross-validated values (“a 5-fold cross-validated AUC-ROC of 0.934 ± 0.026 and an AUC-PR of 0.761 ± 0.091—the two highest values among the four compared classifiers”), described the four classifiers as statistically indistinguishable, and stated that “a comparable performance is preserved on the external IBM HR Analytics benchmark (Section 4.12).” A correction has been made to the Conclusion:

The empirical evidence reported in Section 4 shows that the proposed compact MLP with a 40–128–64–32–2 funnel topology, trained on SMOTE-balanced data with AdamW, cosine annealing and early stopping, attains, under a leak-free cross-validation protocol, a 5-fold cross-validated AUC-ROC of 0.916 ± 0.030 and an AUC-PR of 0.713 ± 0.101, within one cross-fold standard deviation of—and not exceeding—logistic regression, random forest and gradient boosting (gradient boosting attains the highest mean AUC-ROC and AUC-PR; fold-level paired tests are mostly non-significant, while the pooled out-of-fold DeLong test finds the small ROC advantages of gradient boosting and logistic regression statistically significant; Section 4.5). Because the IBM HR Analytics dataset is the source from which the Watson benchmark was derived, it cannot serve as external validation (Section 4.12). The framework is robust to the choice of imbalance-handling strategy among SMOTE, ADASYN, Borderline-SMOTE and class-weighted cross-entropy (Section 4.10), and performs within one cross-fold standard deviation of two modern tabular deep architectures—NODE and FT-Transformer—while clearly outperforming TabNet at this sample size, all trained under the same leak-free protocol (Section 4.8). It is preferred over the gradient-boosting baseline not on raw accuracy grounds—on which gradient boosting holds a small, statistically significant ROC edge of 0.027 AUC-ROC points—but on the methodological grounds developed in Section 4.9, namely the smooth, differentiable attribution semantics of DeepSHAP, the well-calibrated log-odds outputs of the network, and the natural extensibility of the deep computational graph to longitudinal, multimodal real-data inputs.

A fourth limitation has been added to the list of limitations that follows:

(iv) this study performs no economic or cost-effectiveness analysis (no cost, QALY, or incremental cost-effectiveness quantities are estimated), so the identified attrition drivers should be read only as candidate inputs that a subsequent, dedicated economic evaluation on real workforce data could price, and the contribution is methodological rather than health-economic.

The data availability statement described the IBM dataset as an external-validation dataset, which is inconsistent with the corrected Section 4.12. It erroneously read: “The external-validation dataset is the IBM HR Analytics Employee Attrition & Performance dataset, also synthetic, distributed publicly by IBM and widely used as a tabular machine-learning benchmark.” The correct sentence is:

The IBM HR Analytics Employee Attrition & Performance dataset (also synthetic, distributed publicly by IBM) is the source from which the Watson Healthcare benchmark was derived; it is referenced here only to document that provenance and was not used as an independent validation set.

The original version of this article has been updated.

